# Macrophage-organoid co-culture model for identifying treatment strategies against macrophage-related gemcitabine resistance

**DOI:** 10.1186/s13046-023-02756-4

**Published:** 2023-08-09

**Authors:** Shengwei Jiang, Tingwei Deng, Huan Cheng, Weihan Liu, Dan Shi, Jiahui Yuan, Zhiwei He, Weiwei Wang, Boning Chen, Li Ma, Xianbin Zhang, Peng Gong

**Affiliations:** 1 Department of General Surgery & Institute of Precision Diagnosis and Treatment of Digestive System Tumors, Carson International Cancer Center, Shenzhen University General Hospital, Shenzhen University, Shenzhen, Guangdong 518055 China; 2 Guangdong Provincial Key Laboratory for Biomedical Measurements and Ultrasound Imaging, School of Biomedical Engineering, Shenzhen University Medical School, Xueyuan Road 1066, Shenzhen, 518060 China; 3 School of Pharmaceutical Sciences, Health Science Center, Shenzhen University, Shenzhen, 518060 China; 4 Department of Epidemiology, Dalian Medical University, Lvshun Road 9, Dalian, 116044 China; 5 Department of Hepatobiliary Surgery, Henan Provincial People’s Hospital, Weiwu Road 7, Zhengzhou, 450003 China; 6 Carson International Cancer Center & Guangdong Provincial Key Laboratory of Regional Immunity and Diseases, Shenzhen University Health Science Center, Xueyuan Road 1066, Shenzhen, 518060 China

**Keywords:** Gemcitabine resistance, Organoids, Macrophages, Cancer stem cells

## Abstract

**Background:**

Gemcitabine resistance (GR) is a significant clinical challenge in pancreatic adenocarcinoma (PAAD) treatment. Macrophages in the tumor immune-microenvironment are closely related to GR. Uncovering the macrophage-induced GR mechanism could help devise a novel strategy to improve gemcitabine treatment outcomes in PAAD. Therefore, preclinical models accurately replicating patient tumor properties are essential for cancer research and drug development. Patient-derived organoids (PDOs) represent a promising in vitro model for investigating tumor targets, accelerating drug development, and enabling personalized treatment strategies to improve patient outcomes.

**Methods:**

To investigate the effects of macrophage stimulation on GR, co-cultures were set up using PDOs from three PAAD patients with macrophages. To identify signaling factors between macrophages and pancreatic cancer cells (PCCs), a 97-target cytokine array and the TCGA-GTEx database were utilized. The analysis revealed CCL5 and AREG as potential candidates. The role of CCL5 in inducing GR was further investigated using clinical data and tumor sections obtained from 48 PAAD patients over three years, inhibitors, and short hairpin RNA (shRNA). Furthermore, single-cell sequencing data from the GEO database were analyzed to explore the crosstalk between PCCs and macrophages. To overcome GR, inhibitors targeting the macrophage-CCL5-Sp1-AREG feedback loop were evaluated in cell lines, PDOs, and orthotopic mouse models of pancreatic carcinoma.

**Results:**

The macrophage-CCL5-Sp1-AREG feedback loop between macrophages and PCCs is responsible for GR. Macrophage-derived CCL5 activates the CCR5/AKT/Sp1/CD44 axis to confer stemness and chemoresistance to PCCs. PCC-derived AREG promotes CCL5 secretion in macrophages through the Hippo-YAP pathway. By targeting the feedback loop, mithramycin improves the outcome of gemcitabine treatment in PAAD. The results from the PDO model were corroborated with cell lines, mouse models, and clinical data.

**Conclusions:**

Our study highlights that the PDO model is a superior choice for preclinical research and precision medicine. The macrophage-CCL5-Sp1-AREG feedback loop confers stemness to PCCs to facilitate gemcitabine resistance by activating the CCR5/AKT/SP1/CD44 pathway. The combination of gemcitabine and mithramycin shows potential as a therapeutic strategy for treating PAAD in cell lines, PDOs, and mouse models.

**Supplementary Information:**

The online version contains supplementary material available at 10.1186/s13046-023-02756-4.

## Background

Pancreatic adenocarcinoma (PAAD) is one of the most lethal cancers that is poorly understood [1]. While gemcitabine-based chemotherapy is an essential treatment for PAAD [2], resistance to this therapy can lead to poor patient outcomes [3]. Among different types of immune cell infiltration, macrophage infiltration is particularly associated with shorter survival time for PAAD patients [4]. The crosstalk between macrophages and pancreatic cancer cells (PCCs) increases the acquisition of cancer stem-like properties in cancer cells, leading to chemoresistance [5–8]. The relationship between macrophages and cancer cells has been explored in previous studies investigating different crosstalk mechanisms, including those involving microRNAs and exosomes. However, a complete understanding of this relationship remains elusive, leading to the clinical challenge of overcoming gemcitabine resistance (GR). Additionally, despite most drugs being effective in preclinical models, ninety percent fail in clinical drug development [9]. The lack of in vitro preclinical models that accurately replicate patient tumor properties is a major bottleneck in advancing basic cancer research and developing novel cancer therapies [10]. To overcome this, organoids, which are 3D miniature structures cultured in vitro, have been developed as a promising model that can recapitulate the cellular heterogeneity, structure, and functions of human organs. Therefore, in this study, a co-culture model of patient-derived organoids (PDOs) and macrophages was developed to investigate the underlying mechanism of GR and find a promising drug to overcome GR. By analyzing PDOs, 48 PAAD tumor samples, big data, and sequential single-cell transcriptome data, we aimed to elucidate the most likely crosstalk mechanism between macrophages and PCCs that leads to GR. The ultimate aim was to identify a promising combination therapy that can improve gemcitabine treatment and prolong the progression-free survival of PAAD patients.

Cancer stem cells (CSCs) are one factor related to the reduced sensitivity of pancreatic cells to gemcitabine [11], and these cells cause relapse and metastasis by giving rise to new cancers [12]. CD44, a CSC biomarker, is involved in cancer cell proliferation, cell differentiation, cell migration, and angiogenesis [13]. It is also related to GR [14] and regulated by the Sp1 protein [13, 15, 16], a transcription factor [17, 18]. PCCs expressing high levels of CD44 with a mesenchymal-like phenotype are highly invasive and develop GR in vivo [19]. Therefore, we hypothesized that macrophages promoted the acquisition of cancer stem-like properties leading to GR in PAAD via the Sp1/CD44 axis.

Cytokines mediate interactions between cells and have a variety of biological functions, such as regulating cell growth, differentiation, and maturation [20]. CCL5 and AREG are critical cytokines that maintain cancer stem-like properties, such as self-renewal, the potential for nondirectional differentiation, and chemotherapy resistance [21, 22]. Several previous reports have documented the role of CCL5 and AREG in cancer progression. However, the underlying mechanisms of these two cytokines leading to GR remain largely unknown. In this study, we found that CCL5 and AREG formed a positive feedback loop between macrophages and PCCs and further sought to delineate the mechanisms by which the macrophage-CCL5-SP1-AREG loop initiates CSC development and reduces the antitumor activity of gemcitabine via the CCL5/AKT/Sp1/CD44 axis in PAAD.

## Methods

### Tissue specimens

In this study, we employed PAAD tissue specimens obtained from PAAD patients who underwent surgical resections at Henan Provincial Peoples Hospital. All the patients enrolled in this study provided written informed consent. The ethics committee of Henan Provincial Peoples Hospital approved the present study.

### Organoid development and co-culture model in vitro

Organoid development methods followed a previous study [23]. The pancreatic tumor specimens were dissociated into singles cells by 1.0 mg/mL collagenase type I (Sigma-Aldrich). A homemade droplet-based microfluidic system was used to fabricate organoid precursors. The co-culture model in vitro was dependent on transwell inserts. A 0.4 μm transwell was inserted into a 6/24-well plate and divided into two spaces, one for macrophages and another for organoid or pancreatic cell lines. The cell density ratio of macrophages to PCCs was 1:3.

### The Cancer Genome Atlas (TCGA) and The Genotype-Tissue Expression (GTEx) database analysis

TCGA-PAAD data were obtained from the UCSC Xena project(http://xena.ucsc.edu/). First, immune cell infiltration was estimated in 56 patients treated with Gem drugs using CIBERSORT (https://cibersort.stanford.edu/) based on the reported literature. We divided the patients into two groups according to M0 Macrophage level with a cut-off of 0.2, and we fitted and plotted Kaplan‒Meier survival curves using the R package survival and survminer. The differences between the two groups were compared by P values in the log-rank test, and a P value less than 0.05 was considered to indicate statistical significance.

GTEx transcripts per million (TPM) gene expression data were obtained from the UCSC Xena project. The gene expression data were transformed into Log2(TPM + 1) values for further analysis. Differences between normal pancreas (*n* = 171) and tumor (*n* = 179) samples were estimated by one-way ANOVA. A total of 179 PAAD patients were divided into two groups based on gene expression levels compared to lower quartile gene levels.

### Cell viability

Cell viability of PDOs was detected by CellTiter-Glo® Luminescent Cell Viability Assay (Promega). PDOs were cultured for 7 days and transferred into 96 white well plates. 100 µl CellTiter-Glo® reagent per well was added to each well for 15 min. A microplate reader (BIOTEK) was used to record luminescence.

### Flow cytometry

Annexin V-APC/PI was used to analyze the apoptosis level of PANC-1 and 6606PDA cells treated with gemcitabine, MIT, or both for 48 h. Anti-CD44 antibody (Abcam) as a CSC marker was employed to mark PANC-1 cells. APC anti-mouse CD206 antibody (BioLegend) and anti-iNOS AF 488-conjugated antibody (Invitrogen) were used to stain macrophages to identify the type of macrophages. After treatment for 48 h, RAW264.7 cells were harvested and transferred to a 15 ml tube. Antibodies were added at a ratio of 1:500 and incubated at room temperature for 10 min in the dark. Then, 10,000 cells were analyzed.

### Western blotting

Cells were lysed in 100 μl RIPA buffer (MedChemExpress). Proteins were transferred from SDS‒PAGE gels to Immobilon-FLPVDF membranes, blocked, and then incubated with primary antibodies overnight at a 1:1000 dilution. Antibodies against CCR5, AKT, p-AKT, Sp1, CD44, EpCAM, c-Myc, LATS 1, YAP, and GTGF (Abcam) were used. Membranes were then incubated in secondary antibodies, washed, and exposed on a chemiluminescence imaging system (Beijing Sage Creation Science) with ECL (Thermo Fisher Scientific). Western blotting was repeated three times for each band (see Additional file 1).

### Immunofluorescence

After treatment, the cells were fixed with 4.0% PFA for 30 min at room temperature, washed three times, permeabilized with 0.5% Triton X-100 for 5 min at room temperature, incubated in 5% bovine serum albumin for 1 h, and then incubated with antibodies against CD44 (Abcam) at 1:400 overnight. After washing with PBS, the cells were incubated with Alexa Fluor 488 conjugated secondary antibody (Thermo Fisher Scientific). Finally, the nuclei were stained with DAPI (1:1000, Beyotime). The imaging was captured by confocal microscopy.

### Sphere formation

A total of 1000 cells per well were seeded into a 6-well Nunclon Sphera surface dish (Thermo Fisher Scientific). The medium was changed every 3 days. After 15 days of culture, images were captured, and the number of spheres was counted.

### ELISA

The concentrations of CCL5 and AREG were quantified by ELISA (FineTest). The cell supernatant was collected. One hundred microliters per well were added to 96-well plates coated with primary antibody for 90 min. Then, enzyme conjugate solutions were added to all wells. After incubation for 60 min, the plate was washed 6 times. Ninety microliters of TMB substrate were added to all wells for 20 min. Finally, 50 μl of stop solution was added. The absorbance was detected by a microplate reader at 450 nm.

### Animal assay

All animal experiments were approved by the Animal Ethical Committee of Shenzhen TopBiotech Co., Ltd. and performed following the Guidelines for the Care and Use of Laboratory Animals. Adult male mice were purchased from Guangdong Medical Laboratory Animal Center and maintained in the specific pathogen-free Shenzhen TOP Biotechnology Co., Ltd. Laboratory Animal Center.

A 5 μl cell suspension containing 2.5 × 10^5^ 6606PDA cells was transplanted into the pancreas of C57BL/6 J mice (4–6 weeks, male) to construct the orthotopic pancreatic carcinoma mouse model. The mice (*n* = 6) were intraperitoneally administered 50 mg/kg gemcitabine, 0.2 mg/kg MIT [24], or both treatments twice per week. After 5 weeks of treatment, all mice were sacrificed through cervical dislocation after isoflurane anesthesia. The pancreatic tumors were removed and weighed.

Mouse tumor-bearing models were used to observe tumor growth following macrophage stimulation. After co-cultured with RAW264.7 cells for 14 days, 2 × 10^6^ 6606PDA cells in 100 μl were subcutaneously transplanted into nude mice (4–6 weeks old, male). After 7 days, the size of the tumors was measured every 3 days. On day 19, all mice were sacrificed through cervical dislocation isoflurane anesthesia. The pancreatic tumors were removed and weighed.

### Single-cell RNA-sequencing analysis

The data of single-cell RNA- sequencing of mouse pancreas during the progression from preinvasive stages to tumor formation was obtained from Schlesinger et al. [25]study, which has been deposited in NCBI's Gene Expression Omnibusstore (GSE141017). The single-cell RNA- sequencing data of 16 PAAD patients was obtained from the Steele et al. [26] study (GSE155698). We performed the standard analysis procedure. Harmony was used to avoid batch effects.

### Cytokine antibody array (97 targets)

A total of 6 × 10^5^ 6606PDA cells were seeded in 6-well plates. Blank transwells were inserted in the monoculture group, and 2 × 10^5^ RAW 264.7 cells were seeded in transwells in the co-culture model. After 3 days of culture, we collected the culture medium and performed the cytokine antibody array (Abcam) following the manual. First, the membranes containing 96 targets were blocked in a blocking buffer. Two milliliters of culture medium were incubated with the blocked membranes overnight at 4 °C. After washing, 2 mL of Biotin-Conjugated Anti-Cytokines was incubated with membranes overnight at 4 °C. Then, 2 mL of HRP-conjugated streptavidin was added to the washed membranes and incubated overnight at 4 °C. Finally, 500 μL of detection buffer was added to the membranes for 2 min at room temperature. The images were captured by a chemiluminescence imaging system (Beijing Sage Creation Science).

### Gene Expression Omnibus (GEO) analysis

The relevant bigWig and BED data from SP1 ChIP-seq analysis of human K562 cells were downloaded from the GEO database (GSM2424246, GSM2424247), and the images are displayed by igv (version: 2.11.1).

### Construction of cells stably expressing shSp1 and siRNA transfection

ShSp1 PANC-1 cells were generated using the following procedure: 293 T cells at 70% density were utilized for packaging the target plasmid (ShSp1 and NC vector) obtained from GeneChem (Shanghai, China) along with the lentivirus (pMDLg/RRE: Vsvg: pRSV-Rev = 5:3:2). We used the following regions of Sp1 mRNA to design shRNA oligonucleotides: 5’-CCAGGTGCAAACCAACAGATT. Transfection was performed using liposome nucleic acid transfection reagent (YEASEN). The cells were then incubated with these reagents and packaging plasmids for 48 h, and the resulting supernatant was collected. PANC-1 cells at approximately 30% density were cultured 24 h before infection and then incubated with a mixture of complete medium and virus solution at a 1:1 ratio. After subculturing for 48 h, the cells were screened with 2 μg/mL puromycin to select the desired cells. The expression of the target protein (Sp1) was confirmed by Western blotting.

Si-Sp1 transfection: The Sp1 plasmid was purchased from GeneChem (Shanghai, China). The target sequence was as follows: siSP1 5’-GCAACATCATTGCTGCTAT. Transfection of plasmids into cells was performed using Lipofectamine 2000 (Invitrogen) according to the manufacturer's instructions. The expression of the target protein (Sp1) was confirmed by Western blotting.

### Cell culture and macrophage polarization

The human pancreatic cancer cell lines PANC-1 and MIA PaCa-2, as well as the macrophage cell lines THP-1 and RAW264.7, were obtained from the National Collection of Authenticated Cell Cultures (Shanghai, China). The mouse pancreatic cancer cell line 6606PDA was kindly provided by Prof. Tuveson from the University of Cambridege, UK. The cells were cultured in DMEM (HyClone) supplemented with 100 U/mLpenicillin‒streptomycin and 10% fetal bovine serum (FBS) (GIBCO) at 37 °C in a 5% CO_2_ incubator. The cells were not cultured beyond passage 30.

Consistent with previous studies [27, 28], M1-type macrophages were generated by treating the cells with LPS (1000 ng/ml) and IFN-γ (20 ng/ml) for 24 h. Similarly, M2-type macrophages were generated by treating the cells with IL-4 (20 ng/ml) and IL-13 (20 ng/ml) for 24 h.

### Statistical analysis

The data are presented as the mean ± SD. The statistical comparisons between the two groups were conducted with a two-tailed Student's t test by Prism GraphPad 9. A p value less than 0.05 was considered to indicate a significant or highly significant difference.

## Results

### Macrophages are involved in gemcitabine resistance across multiple models: cell lines, PDOs, and clinical data

**Fig. 1:**
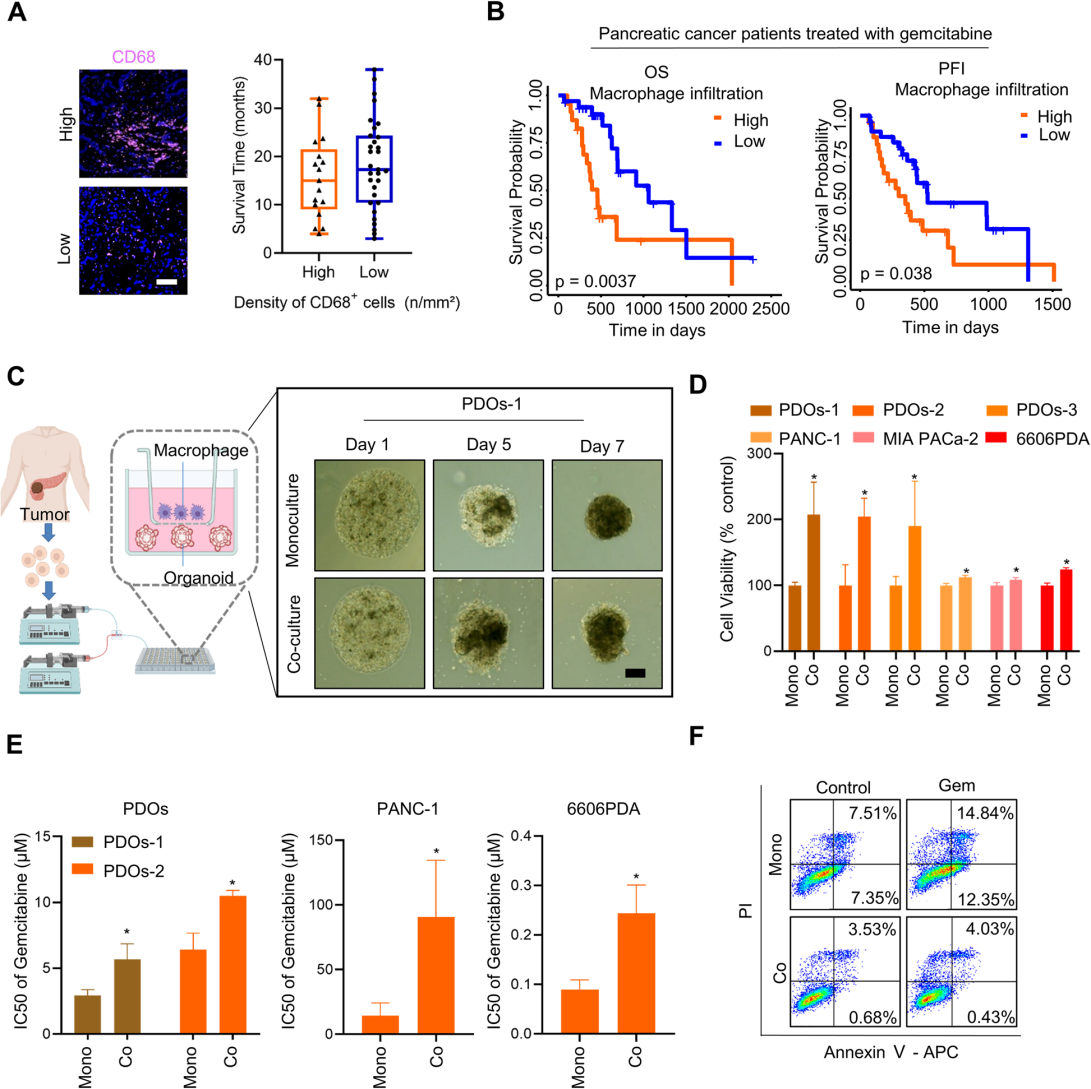
Macrophages induce GR in cell lines, PDOs, and clinical data from PAAD. **A** Representative pictures of PAAD tumor samples stained by CD68 (left); the survival time in different groups (right) (*n* = 48). Scale bar: 100 μm. **B** Overall survival (OS) and progression-free interval (PFI) time of PAAD patients treated with gemcitabine. Data were extracted from The Cancer Genome Atlas (TCGA) (*n* = 56). **C** A schematic diagram of organoid development via the droplet-based microfluidic device. Representative PDO images of the monoculture model and co-culture model. Scale bar: 200 μm. **D** Cell viability analysis of PDOs, human PAAD cell lines (PANC-1, MIA PaCa-2), and a mouse PAAD cell line (6606PDA) in the monoculture model and co-culture model (*n* = 3 ~ 5). **E** Gemcitabine IC_50_ in the mono or co-culture model (*n* = 3 ~ 5). **F** Analysis of apoptosis levels of PANC-1 cells in mono or co-culture model after treatment with 2 μM gemcitabine for 2 days. (*n* = 3). *p < 0.05

To better understand the influence of macrophage infiltration in PAAD, 48 PAAD tumor samples and clinical information were collected. The tumor sections were analyzed using CD68 staining to determine the abundance and distribution of macrophages [29]. The average survival time of PAAD patients with high expression of CD68 was 15.6 months (*n* = 17), compared with 18.4 months (*n* = 31) in the low expression of CD68 group (Fig. 1A). We subsequently analyzed The Cancer Genome Atlas (TCGA) data for 56 PAAD patients who received treatment with gemcitabine. The results showed that high macrophage infiltration significantly reduced overall survival (OS) and progression-free interval (PFI) (Fig. 1B). These findings suggest that macrophage infiltration in PAAD may affect the effectiveness of gemcitabine therapy.

Although the above clinical data demonstrate that macrophage infiltration is related to GR, it is important to conduct additional experiments with a single variable to confirm the impact of macrophages on GR. To this end, we utilized droplet-based microfluidic devices to fabricate PDOs from tumor samples collected from three PAAD patients, as described in previous studies [23, 30, 31]. Next, A co-culture in vitro model was built to study the crosstalk between PCCs and macrophages separated by 0.4 μm Transwell inserts. Representative PDO images from two different culture models were captured on days 1, 5, and 7 (Fig. 1C). The co-culture group exhibited irregular morphology and occupied a significantly larger area than the monoculture group (Supplementary Fig. S1), revealing that the co-culture model was more invasive than the monoculture model. We further examined cell proliferation in both models, analyzing the density of cells from three PDOs and three PAAD cell lines (PANC-1, MIA PaCa-2, and 6606PDA [32]). Our findings indicated that the co-culture model had a higher density of cells (Fig. 1D).

Notably, the half-maximal inhibitory concentration (IC_50_) of gemcitabine was higher in the co-culture model than in the monoculture model (Fig. 1E), suggesting that macrophages are a contributing factor to gemcitabine resistance. 6606PDA cells in both models following treatment with a range of gemcitabine concentrations clearly showed macrophage-induced GR (Supplementary Fig. S2). It is well-known that gemcitabine mainly causes cell death via apoptosis. Consequently, apoptosis was detected by flow cytometry in the two models. The results suggested that macrophages suppress the gemcitabine-induced apoptosis of PCCs (Fig. 1F). Therefore, the results above establish that macrophages lead to GR in PAAD.

### The CCL5/AREG loop between macrophages and PCCs regulates the response to gemcitabine

**Fig. 2:**
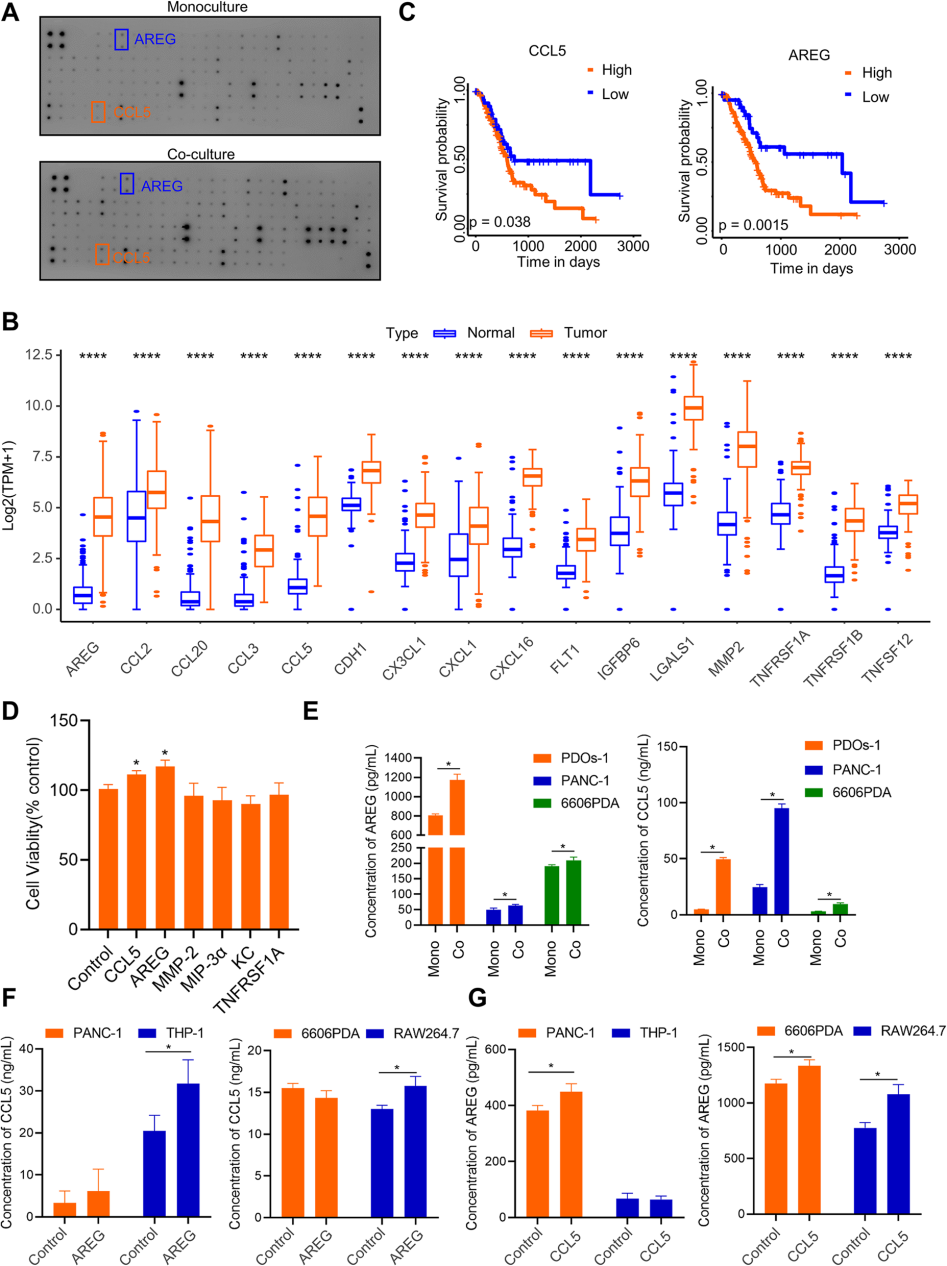
The CCL5/AREG loop allows the communication between macrophages and PCCs. **A** A 97-target cytokine array was used to assess cytokine changes in the mono or co-culture model after 3 days of culture. **B** The gene expression of 16 cytokines in PAAD tumor or normal tissue according to the TCGA and GTEx databases. **C** Kaplan‒Meier survival analysis of CCL5 and AREG in PAAD. **D** Cell viability of 6606PDA cells treated with different cytokines at 100 ng/ml for each cytokine for 3 days. (*n* = 3 ~ 5). **E** The quantitative analysis of AREG (left) and CCL5 (right) by ELISA in different cells of the two models after 3 days of culture. (*n* = 3). **F** Macrophages (RAW264.7 and THP-1 cells) or PCCs (6606PDA and PANC-1 cells) were stimulated with 100 ng/ml AREG for 3 days to induce CCL5 secretion. (*n* = 3). **G** Macrophages and PCCs were stimulated with 100 ng/ml CCL5 for 3 days to induce AREG secretion. (*n* = 3). *p < 0.05

In our in vitro model, macrophages and PCCs shared a culture medium but were separated into two spaces without cell–cell contact. They communicate with each other through chemical signals such as cytokines, exosomes, and metabolites. This study aimed to identify the cytokines that promote GR. To do this, we used a cytokine array containing 97 targets to compare the culture medium of two in vitro models. There were 24 cytokines defined as highly expressed cytokines in the co-culture model (Fig. 2A). TCGA and GTEx transcripts per million (TPM) gene expression data were obtained from the UCSC Xena project to analyze the gene expression of those cytokines in PAAD. One-way ANOVA estimated differences between normal (*n* = 171) and tumor (*n* = 179) samples. 16 of 24 cytokines were highly expressed in PAAD patients (Fig. 2B). In total, 6 of 16 cytokines were associated with a poor prognosis (Fig. 2C and S3). Thus, CCL5, AREG, MMP-2, CCL20, TNFRSF1A, and CXCL1 were identified as potential cytokines responsible for communication between macrophages and PCCs. We further confirmed that CCL5 and AREG stimulated PCC proliferation through a cell viability assay (Fig. 2D). Thus, we focused on CCL5 and AREG in PCCs and macrophages for further investigation.

In the PDO co-culture model, the concentrations of CCL5 and AREG were higher than those in the monoculture model, consistent with both PDO and cell line experiments (Fig. 2E). To determine the primary cells responsible for secreting these cytokines, we stimulated PCCs and macrophages by adding CCL5 or AREG to the culture medium. The results indicated that macrophages likely secreted CCL5 following AREG stimulation (Fig. 2F). Similarly, after CCL5 stimulation, 6606PDA and PANC-1 cells displayed increased expression of AREG (Fig. 2G). However, the secretion of AREG was significantly higher in murine macrophages compared to human macrophages. These results may suggest that CCL5 activates specific signaling pathways or transcription factors in murine cells, leading to the secretion of AREG. However, it appears that this response may not occur in human cells. Therefore, the above data suggested that the CCL5/AREG loop is probably involved in the communication between PCCs and macrophages.

### Macrophages promote the acquisition of cancer stem-like properties in PAAD via CCL5

**Fig. 3:**
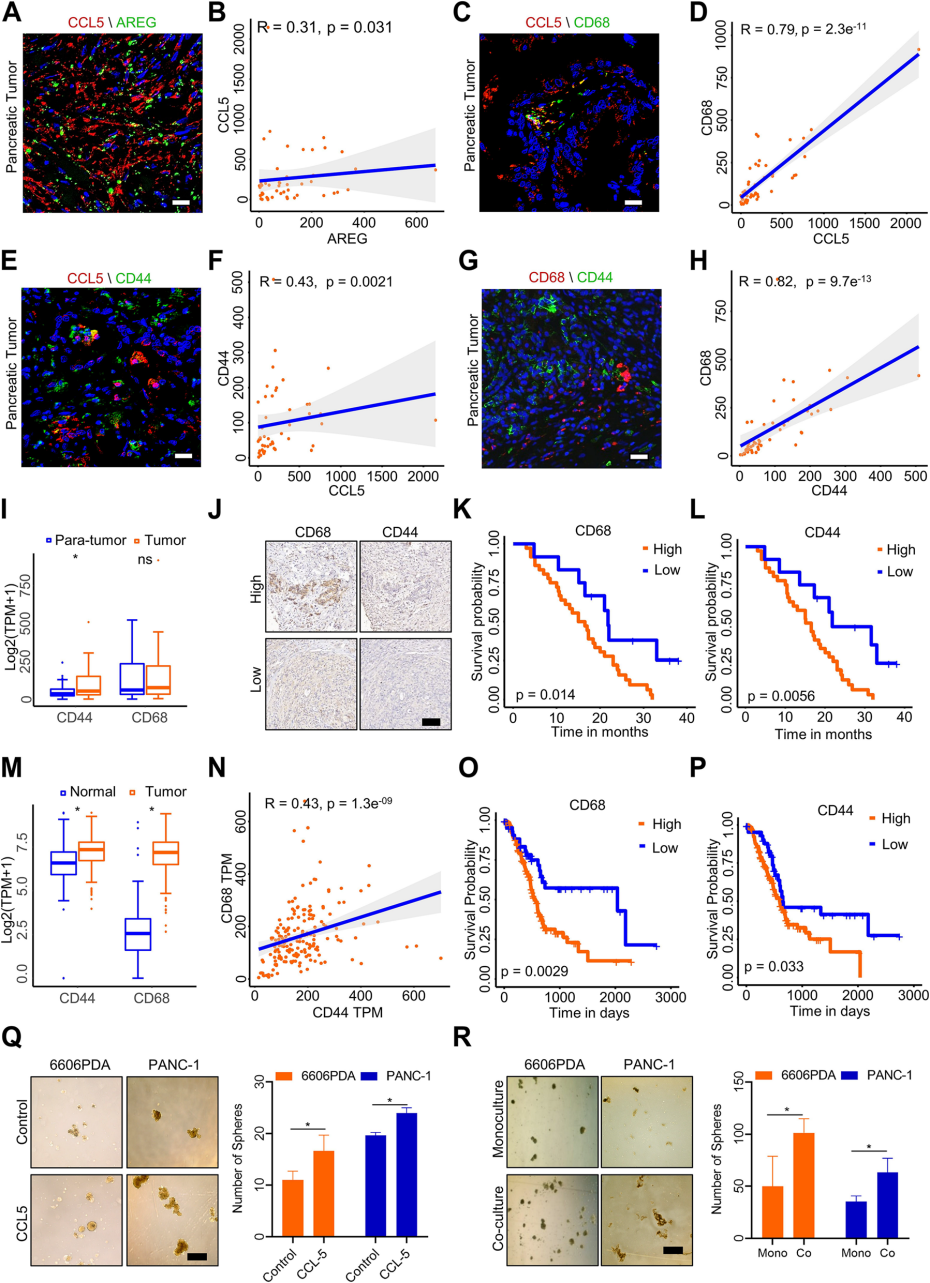
Macrophage-derived CCL5 promotes cancer stemness in PAAD. Representative pictures of CCL5/AREG (**A**), CCL5/CD68 (**C**), CCL5/CD44 (**E**), and CD68/CD44 (**G**) in 48 PAAD tumor samples. Scale bar: 50 μm. Positive density Spearman’s correlation analysis of CCL5/AREG (**B**), CCL5/CD68 (**D**), CCL5/CD44 (**F**), and CD68/CD44 (**H**). (*n* = 48). **I** The positive density of CD44 and CD68 in tumor or para-tumor tissues. (*n* = 48). **J** Representative pictures of CD44 and CD68 in an additional 12 PAAD tumor samples. Scale bar: 100 μm. Kaplan‒Meier survival analysis of CD68 (**K**) and CD44 (**L)** in 48 PAAD tumor samples. **M** Gene expression of CD44 and CD68 in tumor or normal tissue in the TCGA and GTEx databases. **N** The Spearman's correlation analysis of CD44 and CD68. Kaplan–Meier survival analysis of CD68 (**O**) and CD44 (**P**) in the TCGA database. **Q** Representative sphere formation assay images (left) and quantitative analysis (right) of PCCs stimulated with 100 ng/ml CCL5 for 15 days. Scale bar: 200 μm. **R** Representative sphere formation assay images (left) and quantitative analysis (right) of the two models on day 15. Scale bar: 200 μm. (*n* = 3). *p < 0.05

To investigate the role of CCL5 in PAAD, we conducted a comprehensive analysis of 48 tumor sections using various biomarkers. The density of positive cells (number/mm^2^) was calculated. First, we found that CCL5 and AREG were dispersed between cells (Fig. 3A). The correlation coefficient between the density of CCL5-positive cells and AREG-positive cells was 0.31 (Fig. 3B), indicating that CCL5 and AREG were present in the tumor microenvironment. We further observed that CCL5 colocalized with CD68 (Fig. 3C) and had a stronger correlation with CD68 (Fig. 3D), suggesting that CCL5 is secreted by macrophages. Previous studies have shown that macrophages interact with CSCs, contributing to GR [33–35]. To further investigate this relationship, we stained PAAD tumors with CD44, a CSC biomarker, and found that CCL5 also colocalized with CD44 (Fig. 3E), with a correlation coefficient of 0.43 (Fig. 3F). We also found a strong correlation between CD68^+^ cells and CD44^+^ cells (Figs. 3G and H), confirming the role of macrophages in CSC development. Additionally, the positive rate of CD44 staining in tumor sections was higher than that in para-tumor areas (Fig. 3I and S4A). To further elucidate the relationship between macrophage infiltration and CSCs, an additional 12 PAAD tumor samples were collected for CD68 and CD44 immunohistochemical staining (Fig. 3J). The density of positive cells in each section was analyzed (Supplementary Fig. S4B). The correlation between CD68 and CD44 was calculated (Supplementary Fig. S4C), and the correlation coefficient was high at 0.72. Finally, we observed that high positive rates of CD68 and CD44 staining in 48 PAAD patients were associated with shorter survival (Fig. 3K and L). These findings suggest that CCL5, secreted by macrophages, plays a critical role in the development of CSCs and chemoresistance in PAAD.

To increase the sample size, the TCGA and GTEx databases were analyzed. The results showed that CD68 and CD44 were expressed at higher levels compared to normal tissues (Fig. 3M). A moderate correlation was observed between CD68 and CD44 (Fig. 3N), and both markers were associated with a poor prognosis in the 179 PAAD patients (Fig. 3O and P). Thus, a large number of PAAD samples suggest that CD68^+^ macrophages are related to CD44^+^ cells and affect PAAD treatment, indicating a strong relationship between macrophages and CSCs.

Next, mouse tumor-bearing models were used to observe tumor growth following macrophage stimulation. 6606PDA cells were co-cultured with RAW264.7 cells for 14 days and then transplanted into nude mice subcutaneously. The results showed that the tumor volume in the co-culture group was significantly higher than that in the monoculture group (Supplementary Fig. S5A and B). The concentrations of AREG and CCL5 were assessed using serum samples from the two groups. The results indicated that CCL5 levels were significantly increased in the co-culture group (Supplementary Fig. S5C). Tumor sections were stained with CSC biomarkers (CD44, c-Myc, EpCAM) and macrophage biomarkers (F4/80, CD68). In the co-culture model, the tumors highly expressed those biomarkers (Supplementary Fig. S5D). According to hematoxylin–eosin staining, tumors in the co-culture group showed larger areas of focal necrosis (Supplementary Fig. S5D).

In an in vitro model, a sphere formation assay was applied to evaluate the stemness of PCCs after treatment with CCL5 or macrophages. This study revealed that CCL5 increased PAAD stemness (Fig. 3Q). Additionally, PCCs formed many more spheres in the co-culture model, probably owing to macrophage stimulation (Fig. 3R). Therefore, these findings suggest that macrophages promote the acquisition of cancer stem-like properties in PAAD via CCL5.

### Macrophages enhance PCC stemness to resist gemcitabine treatment via the CCL5/AKT/Sp1/CD44 axis

Having established that macrophage-derived CCL5 plays a crucial role in the acquisition of CSC properties that enable resistance to gemcitabine, it is essential to identify the underlying molecular mechanisms and potential therapeutic targets to overcome GR in the clinic. First, we aimed to identify key proteins that could regulate AREG and CSC biomarkers, such as CD44, EpCAM, and c-MYC. To achieve this, we analyzed the Gene Expression Omnibus (GEO) dataset (GSM2424246, GSM2424247) and found that Sp1, a transcription factor, met the requirements (Supplementary Fig. S6A). Previous studies have shown that the AKT signaling pathway is involved in Sp1 regulation. Furthermore, CCR5, present in the cell membrane, is an activator of the AKT signaling pathway [36, 37]. Thus, we hypothesize that macrophages secrete CCL5 to bind to CCR5 in PCCs and then activate the AKT signaling pathway to regulate Sp1 nuclear translocation, ultimately enhancing PCCs stemness.

**Fig. 4:**
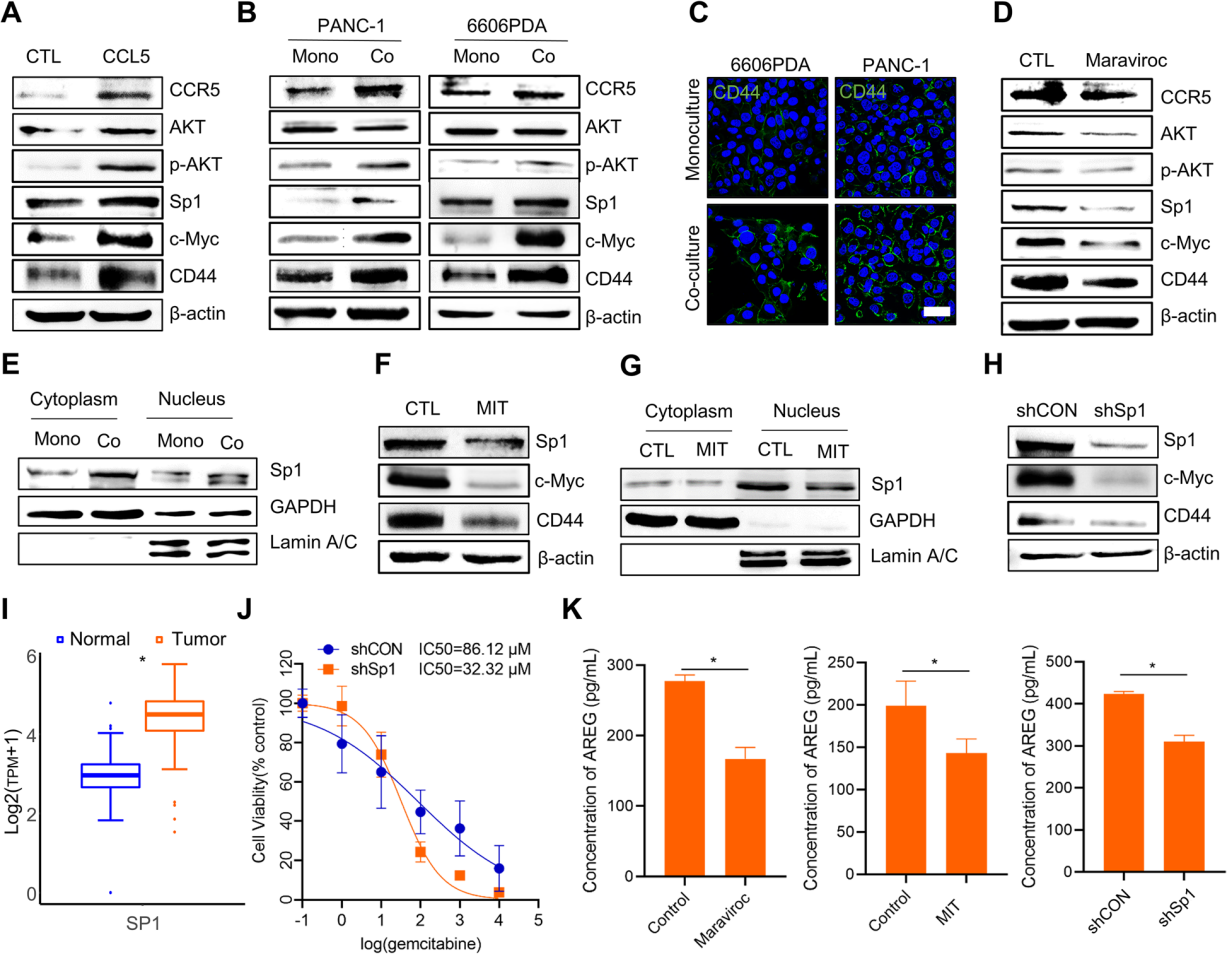
Macrophages enhance PCC stemness via the CCL5/AKT/Sp1/CD44 axis. **A** Protein expression analysis of the CCR5/AKT/Sp1/CD44 axis in PANC-1 cells treated with 100 ng/ml CCL5 for 3 days. **B** In two different models, western blotting analysis of the CCR5/AKT/Sp1/CD44 axis at day 3. **C** Representative immunofluorescence images of CD44 expression. Scale bar: 50 μm. **D** Protein expression of PANC-1 cells treated with 0.75 nM maraviroc, a CCR5 inhibitor, for 2 days. **E** Sp1 nucleus translocation analysis in mono or co-culture model. **F** Protein expression analysis of PANC-1 cells treated with 0.0625 μM mithramycin (MIT) for 2 days. **G** Sp1 nucleus translocation analysis after treatment with 0.0625 μM MIT for 2 days. **H** Western blotting analysis of PANC-1 cells treated with or without shSp1. **I** Gene expression of Sp1 in tumor or normal tissue in the TCGA and GTEx databases. **J** IC_50_ of gemcitabine was determined in PANC-1 cells with or without shSp1 transfection. (*n* = 6). **K** The concentration of AREG in PANC-1 cell culture medium treated with or without 0.75 nM maraviroc, 0.0625 μM MIT or shSp1 for 2 days. (*n* = 3). *p < 0.05

To verify our hypothesis, CCL5 was added to the culture medium to stimulate PCCs. The subsequent detection of protein expression was conducted on the CCR5/AKT/Sp1/CD44 axis (Fig. 4A). Upon utilizing the co-culture model, we observed the activation of the CCR5/AKT/Sp1/CD44 pathway, strongly suggesting that macrophage-secreted CCL5 triggered the activation of the CCR5/AKT/Sp1 pathway in PCCs, enabling them to acquire stem-like properties (Fig. 4B and S6B). We also detected the expression of CD44 (Fig. 4C and S6C) in different cell lines to observe an increase in CD44^+^ PCCs.

Next, CCR5/AKT/Sp1/CD44 pathway inhibitors, small interfering RNA (siRNA), and short hairpin RNA (shRNA) were applied to verify the importance of the CCR5/AKT/Sp1/CD44 pathway in PCCs. Maraviroc, an antiretroviral medication [38], inhibited the CCR5 receptor in the co-culture model. When CCR5 was blocked, the protein expression of AKT, p-AKT, Sp1, c-Myc, and CD44 decreased (Fig. 4D). Mithramycin (MIT) is an antineoplastic antibiotic used to treat testicular cancer, glioma, and Paget's disease of bone [39]. MIT competitively inhibits the binding of Sp1 to its target genes' regulatory elements, such as VEGF, c-Myc, c-Src, XIAP, survivin, and other genes [40]. Moreover, our findings revealed that macrophage stimulation caused Sp1 to translocate into the nucleus (Fig. 4E). MIT was found to decrease the expression of Sp1, c-Myc, and CD44 (Fig. 4F), and MIT reduced Sp1 nuclear translocation (Fig. 4G). Consequently, MIT effectively blocked the Sp1/CD44 pathway as an Sp1 inhibitor.

Sp1 is a ubiquitous transcription factor that plays a critical role in both normal and cancerous biological processes such as cell growth, differentiation, angiogenesis, apoptosis, cellular reprogramming, and tumorigenesis [41]. To investigate its potential for cancer treatment in PAAD, we used siRNA and shRNA technology to silence Sp1. The results were consistent with those obtained using an Sp1 inhibitor, as we observed decreased c-Myc and CD44 expression (Fig. 4H and S6D). In the TCGA-GTEx database, Sp1 was significantly overexpressed in PAAD tumors (Fig. 4I), prompting us to calculate the IC_50_ of gemcitabine in shSp1 PANC-1 cells. Interestingly, we observed an improvement in gemcitabine therapeutic efficacy in PAAD with decreased Sp1 expression (Fig. 4J). Additionally, when blocking the CCR5/AKT/Sp1/CD44 axis with maraviroc, MIT, and shSp1, we observed a corresponding decrease in AREG secretion in PCCs (Fig. 4K) but no significant effect on cell viability (Supplementary Fig. S6E).

Despite the numerous studies conducted on the mechanisms by which macrophage-induced CSCs resist gemcitabine, it is undeniable that GR remains a significant challenge affecting PAAD patient survival in the clinic. Our findings have revealed a novel pathway highlighting the therapeutic potential of targeting the CCR5/AKT/Sp1/CD44 axis to overcome GR.

### PCC-derived AREG promotes CCL5 secretion through the Hippo-YAP pathway

**Fig. 5:**
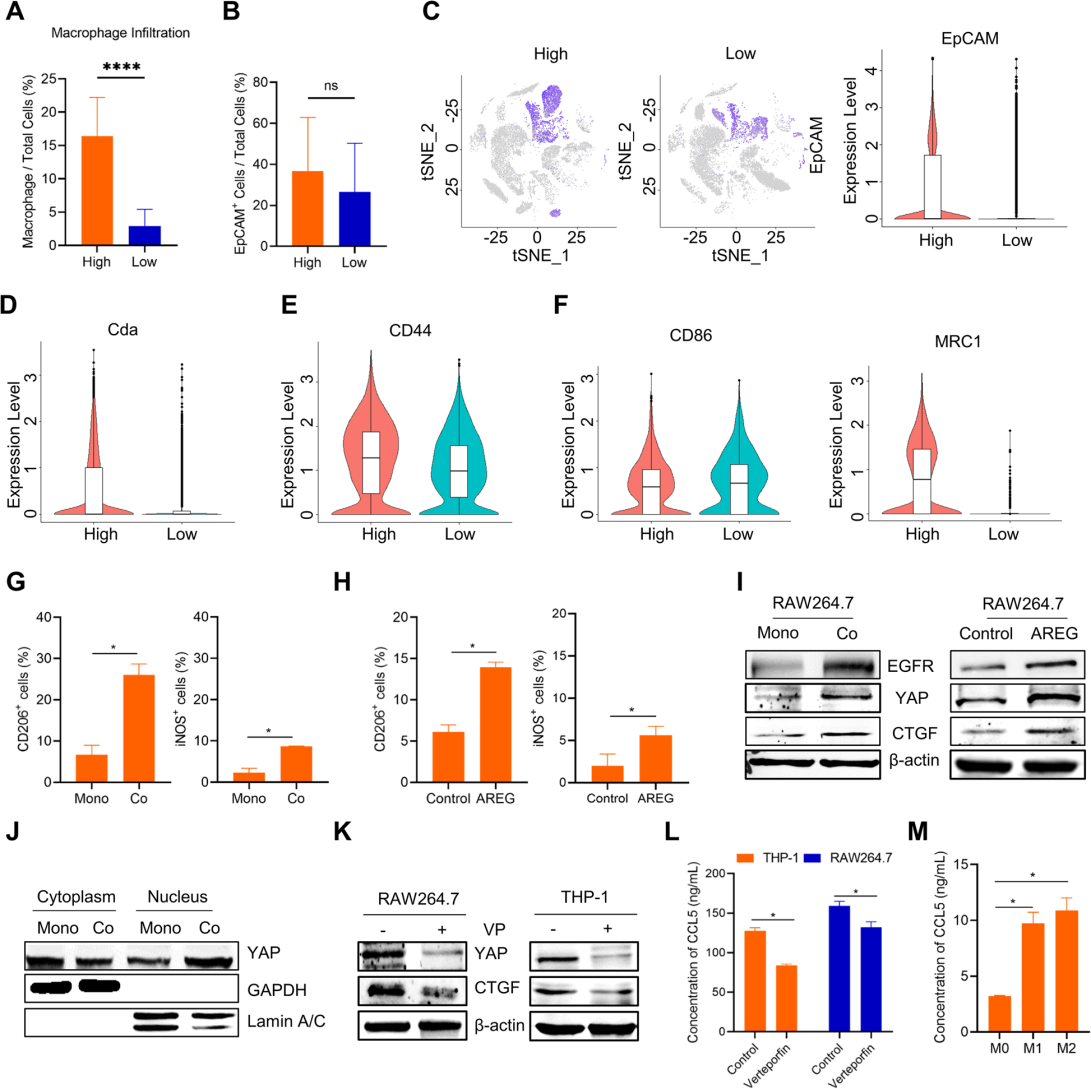
PCC-derived AREG promotes the secretion of CCL5 through the Hippo-YAP pathway. **A** The percentage of macrophage infiltration in 16 PAAD patients. The single-cell sequencing data were obtained from NCBI's Gene Expression Omnibus store (GSE155698). High = 7 samples, Low = 9 samples. **B** The percentage of EpCAM^+^ cells among all cells except for macrophages in the two groups. **C** Gene expression of EpCAM in total cells. **D** Expression of Cda in EpCAM^+^ cells. **E** Expression of CD44 in EpCAM.^+^ cells. **F** Expression of CD86 and MRC1 in macrophages. **G** The types of RAW264.7 cells co-cultured with 6606PDA cells were identified by flow cytometry. CD206 was used to mark M2-type macrophages, and iNOS was used to mark M1-type macrophages. **H** The types of RAW264.7 cells treated with 100 ng/ml AREG for 3 days were identified by flow cytometry. **I** Protein expression analysis of EGFR, YAP and CTGF in RAW264.7 cells following 6606PDA cell or 100 ng/ml AREG stimulation for 3 days. **J** YAP nucleus translocation analysis in two models. **K** Treatment with 0.5 μM verteporfin for 2 days inhibited the Hippo-YAP pathway in macrophages in the in vitro model. **L** Verteporfin reduced macrophage secretion of CCL5. (*n* = 3). **M** The concentration of CCL5 in M0, M1, and M2 type macrophage culture medium. (*n* = 3). *p < 0.05

To investigate the effect of PCCs on macrophages, we conducted an analysis of single-cell sequencing data from 16 PAAD patients in the GEO database (GSE155698). The samples were classified into two groups based on the percentage of macrophage infiltration: high macrophage infiltration (7 samples) and low macrophage infiltration (9 samples) (Fig. 5A). EpCAM, a transmembrane glycoprotein that is highly expressed in various types of cancer and has been identified as a tumor marker of epithelial origins for nearly four decades [42], was used to identify tumor cells in this study. The mean rate of EpCAM^+^ cells among total cells except for macrophages was 36.6% in the high group compared to 26.5% in the low group (Fig. 5B). The expression of EpCAM was higher in the high group than in the low group (Fig. 5C), suggesting that macrophage infiltration promotes tumor cell proliferation, as seen in the survival data for PAAD patients in Fig. 1B. EpCAM^+^ cells were subsequently isolated and analyzed. Cytidine deaminase (Cda), a GR-related gene that inactivates gemcitabine by deamination, was found to be overexpressed in EpCAM^+^ cells in the high macrophage infiltration group (Fig. 5D), indicating resistance to gemcitabine. The expression level of CD44 was also elevated in the high macrophage infiltration group (Fig. 5E), consistent with previous findings shown in Fig. 3. Next, we investigated the type of macrophages present in the two groups. CD86, a biomarker of M1-type macrophages, showed little difference between the two groups, while MRC1, a biomarker of M2-type macrophages, was highly expressed in the high group, suggesting that macrophages mostly transition into the M2 type (Fig. 5F).

To confirm the results of single-cell sequencing data analysis, we identified the types of macrophages by flow cytometry. M2-type macrophages were labeled using CD206, while M1-type macrophages were labeled using iNOS. In the co-culture model, M0 macrophages mainly converted into M2-type macrophages (Fig. 5G). Then, we found that AREG caused M0-macrophages to differentiate into either M1- or M2-type macrophages. However, there was a higher ratio of M2-type macrophages than M1-type macrophages following AREG stimulation (Fig. 5H). M2-type macrophages, also known as tumor-associated macrophages, promote an immunosuppressive tumor microenvironment, which enables cancer cells to evade immune cell attacks and resist chemotherapy.

AREG is a member of the EGF family and is involved in cell proliferation, differentiation, migration, and survival [43]. EGFR serves as the receptor for AREG [44], and we detected high expression of EGFR in macrophages following PCCs and AREG stimulation (Fig. 5I). Activated EGFR regulates the Hippo-YAP pathway, which is a mechanism for macrophage polarization [45]. Previous research has demonstrated that high expression of YAP can polarize macrophages to the M2-like phenotype [46]. Connective tissue growth factor (CTGF), a well-known YAP target gene, was used to confirm high YAP expression [47]. This study found that 6606PDA cells induced RAW264.7 cells to express YAP and CTGF, suggesting that the YAP signaling pathway was activated (Fig. 5I). Likewise, AREG activated the Hippo-YAP pathway (Fig. 5I). Next, the nucleus proteins were separated from the total proteins. The results showed that a portion of the YAP protein was transferred into the nucleus (Fig. 5J). Verteporfin (VP), a YAP inhibitor, was used to evaluate the function of the YAP pathway in PAAD. Treatment of RAW264.7 and THP-1 cells with verteporfin downregulated the YAP signaling pathway (Fig. 5K). It suppressed the secretion of CCL5 from macrophages (Fig. 5L), suggesting that the YAP signaling pathway was involved in the secretion of CCL5 from macrophages. To determine the primary macrophage types that secrete CCL5, we induced M0 macrophages to differentiate into M1 and M2 phenotypes using IFN-γ and LPS, IL4 and IL13, respectively, following previous studies [27, 28] (Supplementary Fig. S7). Elevated levels of CCL5 secretion were observed in both M1 and M2 macrophages compared to M0 macrophages. However, there was no significant difference in CCL5 secretion between the M1 and M2 phenotypes (Fig. 5M). In summary, macrophage infiltration promoted tumor development. Tumor cells mostly stimulated macrophages to transform into the M2 type. PCC-derived AREG promoted macrophages to secrete CCL5 via the Hippo-YAP pathway.

### The macrophage-CCL5-Sp1-AREG feedback loop promotes GR-related gene expression during PAAD development

**Fig. 6:**
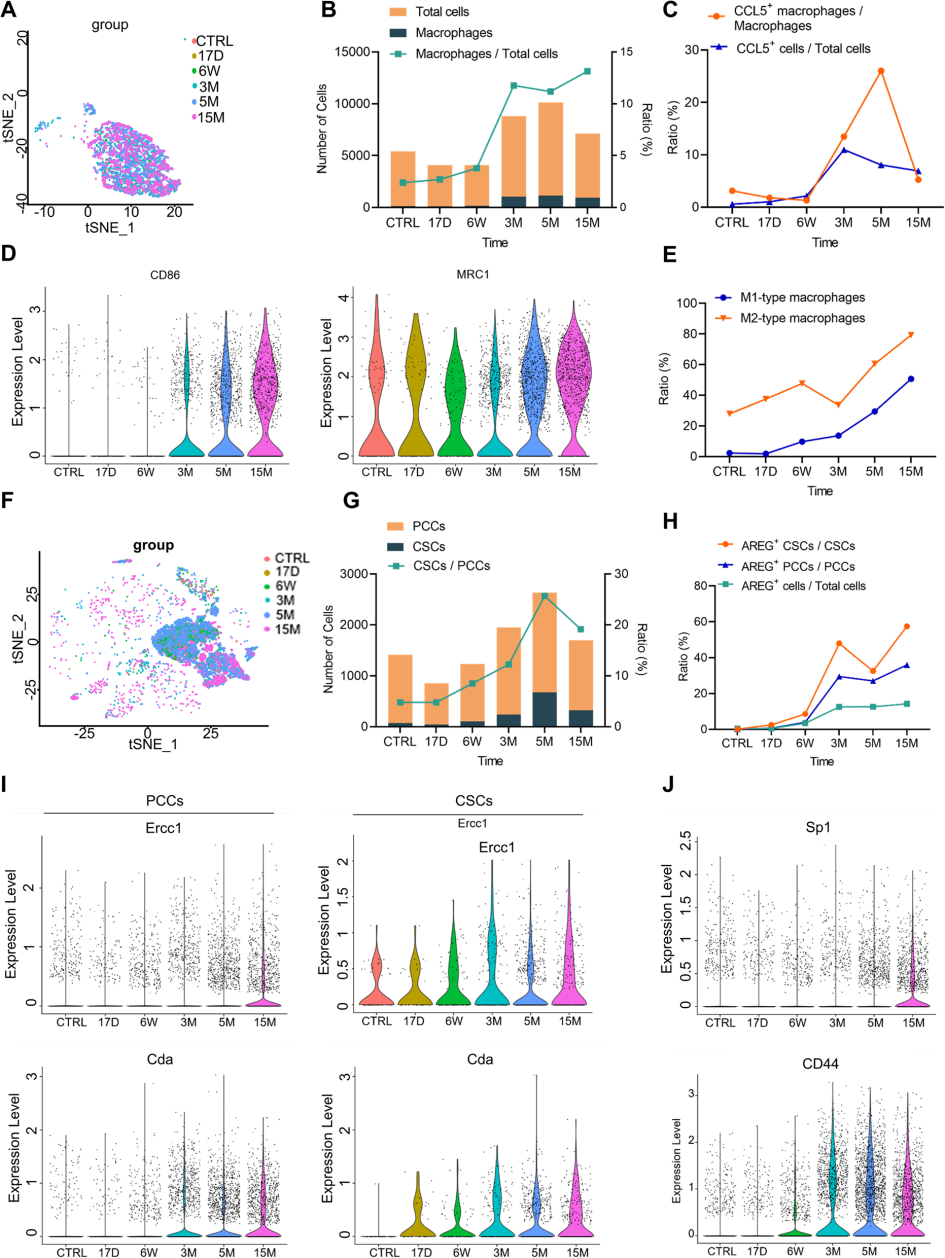
Sequential single-cell transcriptome analysis of mouse pancreatic cancer development. The data were obtained from NCBI's Gene Expression Omnibus repository (GSE141017). **A** Analysis of macrophages. **B** The ratio of macrophages to total cells in different tumor stages. **C** The proportion of CCL5^+^ cells in macrophages and total cells. **D** The expression levels of CD86 and MRC1 in macrophages. **E** The proportion of M1 or M2-type macrophages among total macrophages with tumor progression. **F** Analysis of the tumor cells. **G** The percentage of CSCs to PCCs in different tumor stages. **H** The proportion of AREG^+^ cells among CSCs, PCCs and total cells. **I** The expression levels of the gemcitabine-related genes Ercc1a and Cda in PCCs and CSCs. **J** The expression levels of Sp1 and CD44 in PCCs

Schlesinger et al. [25] performed single-cell RNA sequencing of the mouse pancreas during the progression from preinvasive stages to tumor formation. We analyzed these data (GSE141017) again to verify our hypothesis. The single-cell RNA-sequencing data from control (CTRL), 17 days (17D), 6 weeks (6W), 3 months (3 M), 5 months (5 M), and 15 months (15 M) mouse pancreas samples were selected to analyze the relationship between macrophage infiltration and CSCs. First, 3498 macrophages were isolated from different periods of PAAD development (Fig. 6A). Macrophage infiltration increased with cancer progression, from 2.39% in the CTRL group to 13.14% in the 15 M group (Fig. 6B), suggesting that macrophages were recruited to the tumor microenvironment to support tumor development. CCL5 gene expression in macrophages also increased with tumor progression, which confirmed that macrophages secreted more CCL5 in the middle and advanced disease stages (Fig. 6C). We further analyzed the types of macrophages present during the 15 months and found increased expression of biomarkers for both M1 and M2 macrophages (Fig. 6D). CD86^+^Tir2^+^Il1b^+^ macrophages were defined as M1-type macrophages. MRC1^+^Csf1r^+^ macrophages were M2 macrophages. M0-type macrophages were polarized into M1 or M2 types as tumors developed (Fig. 6E), which supports our previously presented data (Fig. 5G and H).

Furthermore, 9776 EpCAM^+^ cells were defined as tumor cells (Fig. 6F). Within this population, 1452 cells were classified as CSCs that were positive for both CD44 and c-Myc. The changes in the ratio of CD44^+^c-Myc^+^EpCAM^+^ cells to EpCAM^+^ cells over time were investigated. The results demonstrated that as pancreatic tumor development progressed, the level of CSCs increased (Fig. 6G). However, the level of CSCs decreased at 15 months, which may have been due to a reduction in the number of CCL5^+^ macrophages. The changes in the levels of CCL5^+^ macrophages were consistent with the curve of the CSC ratio (Fig. 6C), suggesting that macrophage-derived CCL5 may be associated with the presence of CSCs. Similarly, the increase in the AREG^+^ PCC ratio was consistent with the curve of macrophage increase. (Fig. 6H and B).

To investigate the relationship between CSCs and GR, the study conducted a further analysis of the expression of the GR-related genes Ercc1 and Cda, in CD44^+^c-Myc^+^EpCAM^+^ cells and EpCAM + cells. The results showed higher expression levels of both genes in CD44^+^c-Myc^+^EpCAM^+^ cells, indicating that CSCs were more resistant to gemcitabine with tumor development (Fig. 6I). The study hypothesized that macrophages promote GR in PAAD CSCs, and the single-cell sequencing data confirmed this hypothesis by demonstrating that macrophage infiltration, the CSC ratio, and GR-related gene expressions increased with tumor progression. Further analysis of the CCL5/Sp1/CD44 axis in EpCAM^+^ cells revealed that this signaling pathway was more activated in advanced tumor stages, which was consistent with the protein analysis (Fig. 6J). As a result, potential therapeutic targets were identified among the protein involved in the macrophage-CCL5-Sp1-AREG feedback loop. Disrupting this loop may decrease the proportion of CSCs in the advanced stage, ultimately suppressing GR and improving outcomes for patients with PAAD who are undergoing gemcitabine treatment.

### Targeting the macrophage-CCL5-Sp1-AREG feedback loop enhances the antitumor effects of gemcitabine in PAAD

**Fig. 7:**
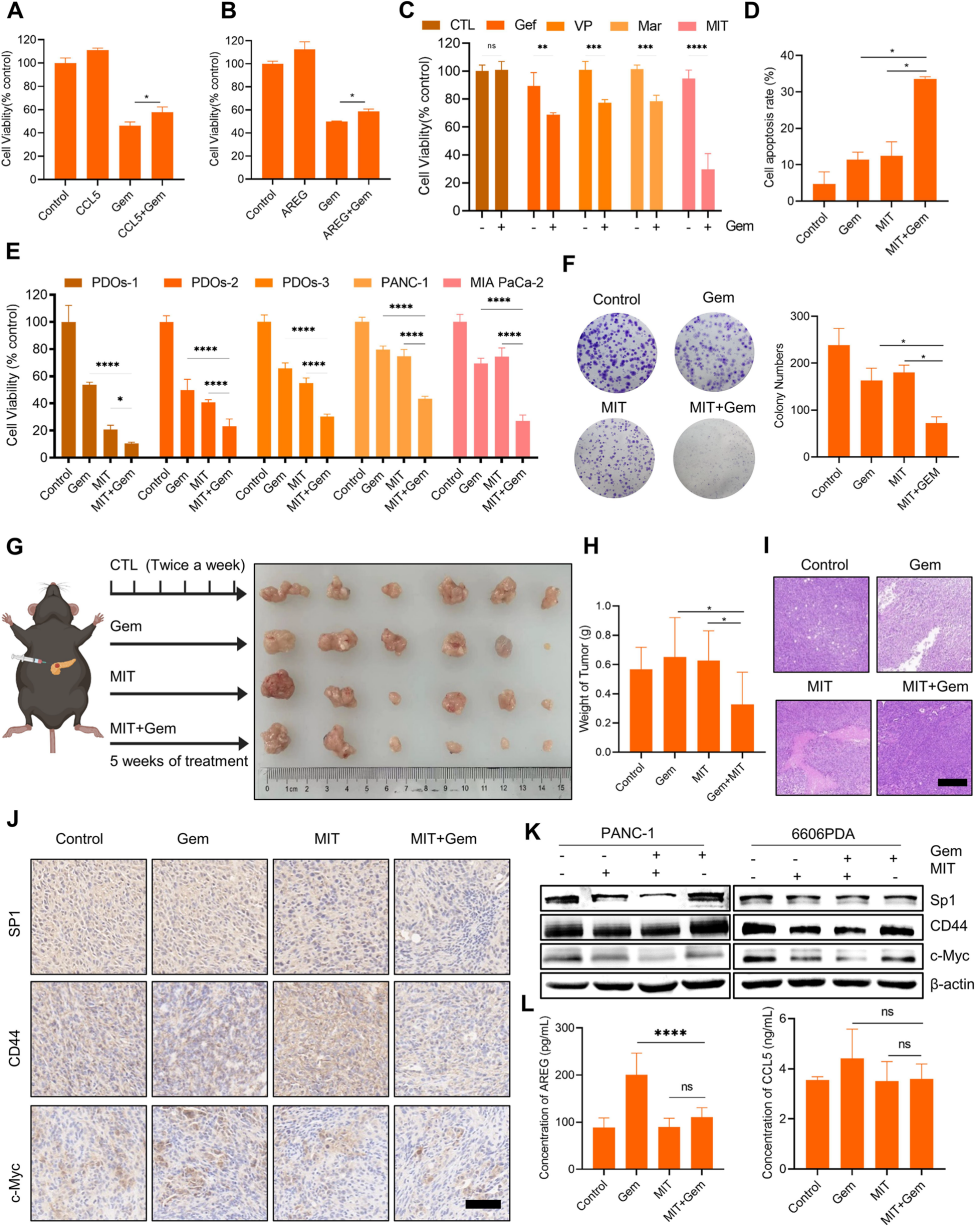
Targeting the macrophage-CCL5-Sp1-AREG loop alleviates GR in the in vivo and in vitro models. **A** The cell viability of 6606PDA cells treated with 0.25 μM Gem and 100 ng/mL CCL5 for 2 days. (*n* = 3). **B** The cell viability of 6606PDA cells treated with 0.25 μM Gem and 100 ng/mL AREG for 2 days. (*n* = 3). **C** The cell viability of 6606PDA cells treated with 0.0625 μM Gem with or without 0.1 μM Gefitinib (Gef); 0.5 μM Verteporfin (VP); 1.5 nM Maraviroc (MAR); or 0.0625 μM Mithramycin (MIT) for 2 days. (*n* = 5). **D** Analysis of apoptosis levels of PANC-1 cells after treatment with 2 μM gemcitabine, 0.0625 μM MIT, or both for 2 days. (*n* = 3). **E** Cell viability of PDOs, PANC-1, and MIA PaCa-2 cells treated with 2 μM gemcitabine, 0.25 μM MIT, or both for 2 days. (*n* = 3 ~ 5). **F** Representative pictures of the colony formation assay (left) and quantitative analysis of colonies (right) (*n* = 3). **G** Image of tumors from the orthotopic pancreatic carcinoma mouse model after treatment with gemcitabine, MIT, or both for 35 days. **H** The tumor weight in different treatment groups. (*n* = 6 tumors). **I** Representative images of HE staining. Scale bar: 50 μm. **J** Representative images of immunohistochemical staining. Scale bar: 50 μm. **K** Protein expression analysis of Sp1, CD44, and c-Myc. **L** The concentrations of AREG and CCL5 in mouse serum. (*n* = 6). *p < 0.05

To investigate potential therapeutic targets in the macrophage-CCL5-Sp1-AREG feedback loop, a series of inhibitors were utilized in combination with gemcitabine to treat PAAD. CCL5 and AREG are signaling factors between macrophages and PCCs, and excessive levels of these cytokines were found to compromise the antitumor effects of gemcitabine (Figs. 7A and B). The concentration of gemcitabine also plays a role in its efficacy, as it was effective at 0.25 μM but ineffective at 0.0625 μM for 6606PDA cells (Supplementary Fig. S8). To further evaluate the treatment effects of gemcitabine combined with different inhibitors targeting the macrophage-CCL5-Sp1-AREG feedback loop, 6606PDA and RAW264.7 co-culture models were employed. The inhibitors included gefitinib (Gef), an EGFR inhibitor targeting the receptor of AREG; verteporfin (VP), a YAP inhibitor; maraviroc (MAR), a CCR5 inhibitor targeting the receptor of CCL5; and mithramycin (MIT), an Sp1 inhibitor. The single drug groups chose a concentration less than the 10% inhibitory concentration. The results showed that while gemcitabine alone did not significantly affect cell proliferation at low concentrations, its combination with macrophage-CCL5-Sp1-AREG feedback loop inhibitors, particularly with MIT, caused significant cell inhibition, indicating that targeting this feedback loop enhances the antitumor effects of gemcitabine in PAAD (Fig. 7C).

The inhibition of Sp1 demonstrated a promising impact on enhancing the effectiveness of gemcitabine treatment. However, previous preclinical studies of Sp1 inhibitors combined with gemcitabine to treat PAAD are limited [48]. Therefore, we evaluated this combination therapy in PAAD using the PDO model, PAAD cell lines, and an orthotopic pancreatic carcinoma mouse model. Our findings showed that MIT significantly increased apoptosis induced by gemcitabine (Fig. 7D) in PANC-1 cells, which are considered a GR cell line. The therapeutic effects of MIT and gemcitabine were further evaluated in three PDOs and two PAAD cell lines. The results demonstrated that MIT is a good choice for improving the outcome of gemcitabine treatment in the in vitro model (Fig. 7E). The colony formation assay clearly showed therapeutic effects in the combination group (Fig. 7F). To further verify the effectiveness of combination therapy, 6606PDA cells were transplanted into the pancreas of C57BL/6 J mice to construct an orthotopic pancreatic carcinoma mouse model [49]. After 35 days of treatment, the cancer size and weight of the combination group were significantly smaller than those of the single drug groups (Figs. 7G and H). The tumor interior in the combination group was denser than that in the single drug groups (Fig. 7I). Immunohistochemical staining showed that the protein expression of Sp1, CD44, and c-Myc was downregulated in the combination group (Fig. 7J), and these results were also observed in the cell line models (Fig. 7K). In mouse serum, the concentrations of CCL5 and AREG were detected, and AREG was found to decrease following treatment with MIT, indicating that MIT inhibits AREG secretion (Fig. 7L).

In conclusion, targeting the macrophage-CCL5-Sp1-AREG feedback loop has the potential to be a therapeutic strategy to improve outcomes for PAAD patients with GR. An optimal option for this purpose is the use of MIT as an Sp1 inhibitor, which can effectively enhance the antitumor effectiveness of gemcitabine. This is demonstrated through the successful disruption of the macrophage-CCL5-Sp1-AREG feedback loop in various models, including cell lines, PDOs, and orthotopic pancreatic carcinoma mouse models.

## Discussion

Gemcitabine is currently the preferred first-line therapy for PAAD, but unfortunately, GR remains a major factor contributing to poor patient outcomes [50–52]. GR can arise either from inherent characteristics of the cancer cells themselves or from the tumor microenvironment [53]. Macrophage infiltration in the tumor microenvironment directly inhibits GEM-induced apoptosis by downregulating caspase-3 activation to promote GEM resistance [54]. PAAD with high macrophage infiltration is associated with shorter OS and PFI. Therefore, it is necessary to devise strategies to improve the efficacy of gemcitabine treatment against resistance induced by macrophages and extend patient survival.

Although numerous strategies show promise in reversing GR in the laboratory, there is a gap between basic research and clinical application, resulting in little effect in the clinic. However, the PDO model is a promising in vitro model to bridge the gap between preclinical and clinical testing, as it can effectively recapitulate the cellular heterogeneity, structure, and functions of human organs, resulting in highly accurate drug predictions. In this study, a droplet-based microfluidic plate was applied to fabricate PDOs to investigate the crosstalk between macrophages and PCCs. We found that macrophages reduced the antitumor activities of gemcitabine, and CCL5 and AREG were identified as the key signaling factors between macrophages and PCCs in the macrophage-organoid co-culture model.

AREG, a member of the epidermal growth factor family, participates in tissue repair and inflammation regulation. Previous studies reported that macrophages secreted AREG to maintain tissue homeostasis [55]. However, in this study, the expression of AREG increased in pancreatic cancer cells along with tumor development, as revealed by analyzing sequential single-cell transcriptome analysis of mouse pancreatic cancer development. Furthermore, in the GEO database and macrophage type identification assays, we found that PCC-derived AREG polarizes macrophages into the M1- or M2-type and induces CCL5 secretion from macrophages by activating the Hippo-YAP signaling pathway. CCL5, a target gene involved in NF-B activity, is expressed by T lymphocytes, macrophages, platelets, synovial fibroblasts, tubular epithelium, and certain types of tumor cells. CCR5 binds with high- affinity to CCL5, CCL3 (MIP-1a), and CCL4 (MIP-1b) in cancer cell membranes to mediate diverse signaling cascades in response to its ligands [56]. The CCL5/CCR5 axis contributes to cancer cell proliferation, metastasis, and the formation of an immunosuppressive microenvironment via the PI3K/Akt or STAT3 signaling pathway [57].

The macrophage-organoid co-culture model revealed CCL5 as a crucial signaling factor involved in the communication between macrophages and PCCs. Subsequent investigations were conducted to gain a deeper understanding of the underlying mechanism. Analysis of clinical information and tumor sections from 48 PAAD patients showed that macrophage-derived CCL5 promotes the acquisition of cancer stemness, which is involved in GR [58]. CSCs are known to possess the ability to self-renew and differentiate into diverse cancer cell lineages, making them resistant to chemotherapy agents such as gemcitabine [58]. In this study, macrophage-derived CCL5 activated AKT pathway signaling in PCCs, subsequently leading to the upregulation of Sp1 protein, which transcriptionally regulated the expression of CD44, c-Myc, and AREG. CD44 and c-Myc serve as biomarkers for CSCs associated with gemcitabine resistance. Analysis of the TCGA and GTEx databases revealed high expression of CD44 in PAAD tissues, displaying a moderate correlation with CD68, a macrophage biomarker. Consequently, macrophages secreted CCL5 to initiate CSC formation by activating the CCR/AKT/Sp1/CD44 axis in PCCs. It was determined that the macrophage-CCL5-Sp1-AREG loop played a critical role in mediating communication between macrophages and PCCs, ultimately leading to the development of gemcitabine resistance.

Our study aimed to uncover viable therapeutic targets and explore combination drugs capable of reversing GR by gaining insight into the mechanism of macrophage-related GR. In the co-culture model, inhibitors of the macrophage-CCL5-Sp1-AREG feedback loop, including Gef, VP, MAR, and MIT, significantly enhanced the treatment effects of gemcitabine in PAAD. Previous studies have demonstrated favorable synergistic effects of gemcitabine and gefitinib in various cancers, such as advanced transitional cell carcinoma and head and neck carcinoma [59, 60]. VP, a recently discovered autophagy inhibitor, effectively blocks autophagy at an early stage by inhibiting the formation of autophagosomes. Donohue et al. demonstrated that VP moderately enhances the antitumor activity of gemcitabine in PAAD [61]. MAR, a CCR5 antagonist, significantly inhibits tumor cell proliferation in PAAD and Hodgkin lymphoma [62, 63]. MIT exhibits potent antitumor activity by inhibiting Sp1 through distinct mechanisms in PAAD, ovarian cancer, and advanced testicular carcinoma [40, 64]. Dauer et al. [48] verified that MIT overcomes gemcitabine-induced chemoresistance in vivo. However, the exact mechanism behind this phenomenon remains elusive and thus warrants further investigation and exploration. One potential avenue to explore is the disruption or inhibition of the macrophage-CCL5-Sp1-AREG feedback loop. Additionally, the clinical use of MIT has been limited due to numerous toxic side effects. To address this challenge, normal tissue organoids are a promising model for screening the effects of MIT and its analogs, nanodelivery systems, and combination therapies. Such screening efforts aim to identify strategies that exhibit lower toxicity profiles, thus offering the potential for enhanced clinical applicability.

By employing a macrophage-organoid co-culture model, we have made significant progress in identifying MIT as a promising drug candidate for enhancing the effectiveness of gemcitabine treatment. To further validate the reliability of the macrophage-organoid co-culture model, we conducted experiments involving three different pancreatic tumor cell lines and an orthotopic pancreatic carcinoma mouse model. These experiments provided additional evidence to support the accuracy and relevance of the macrophage-organoid co-culture model, demonstrating its value as an in vitro model for understanding tumor development mechanisms and predicting potential therapeutic interventions.

## Conclusions

**Fig. 8:**
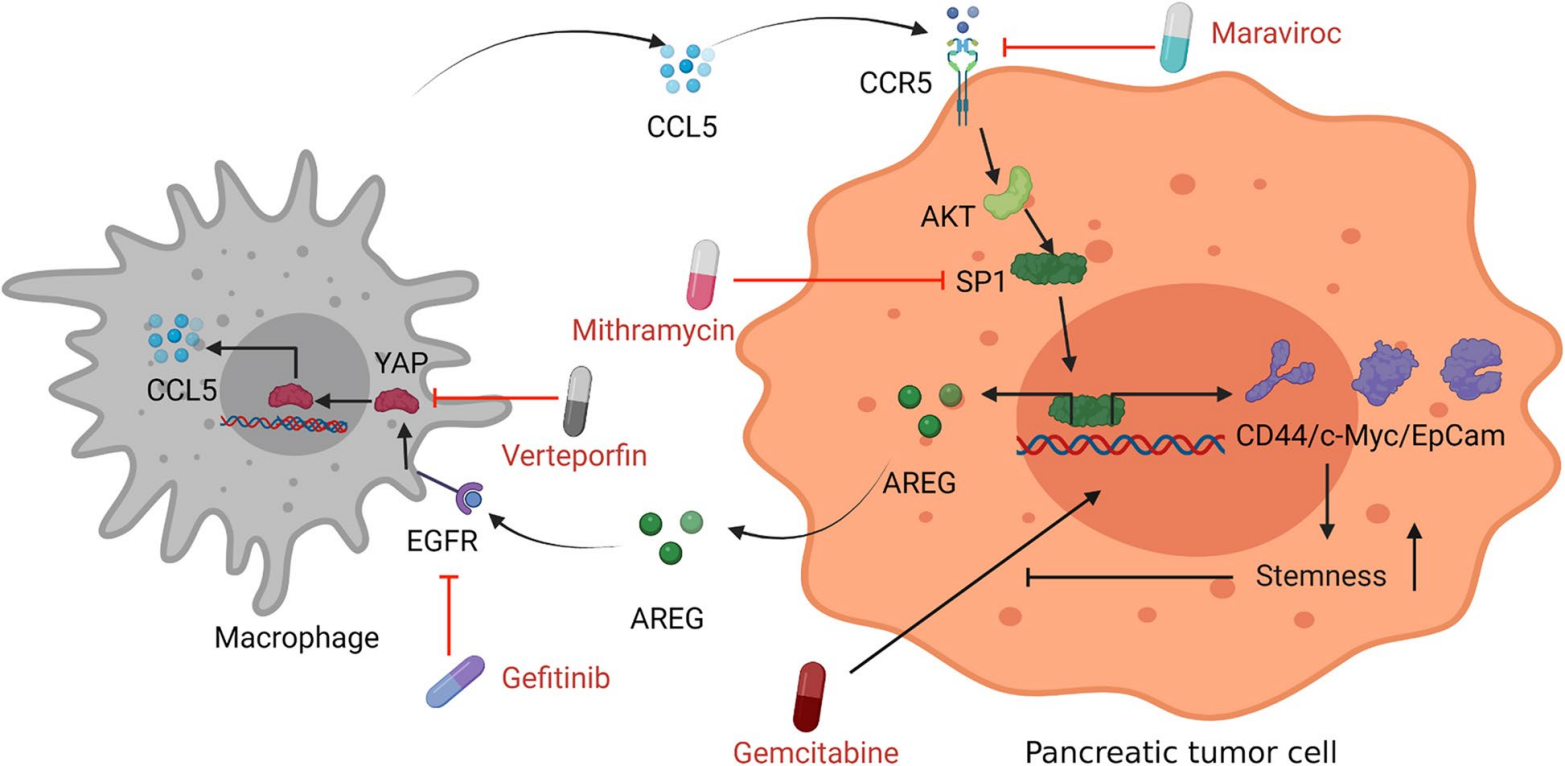
Schematic diagram illustrating that targeting the macrophage-CCL5-SP1-AREG loop between macrophages and PCCs reduces cancer stem-like properties and GR in PAAD. Macrophage-derived CCL5 activates the CCR5/AKT/Sp1/CD44 axis in PCCs, acquiring chemoresistance and secretion of AREG. In response, PCCs produce AREG, which triggers the Hippo-YAP signaling pathway in macrophages, leading to the secretion of CCL5. Therefore, the macrophage-CCL5-Sp1-AREG loop is formed between macrophages and PCCs in the microenvironment. Targeting this loop with drugs like maraviroc, mithramycin, gefitinib, and verteporfin could improve chemotherapy, such as gemcitabine treatment. The PDO model is a superior choice for tumor microenvironment studies and preclinic drug screening. https://biorender.com/

In conclusion, our study highlights the superiority of the macrophage-organoid (PDO) model as an invaluable tool for preclinical research and precision medicine. The findings highlight the significance of utilizing this model to gain insights into complex biological processes and to facilitate the development of personalized therapeutic approaches. Using this model, we elucidated the involvement of the macrophage-CCL5-Sp1-AREG loop in the interplay between macrophages and PCCs. This loop promotes the acquisition of cancer stem-like properties and impedes the antitumor efficacy of gemcitabine in PAAD by activating the CCL5/CCR5/Sp1/CD44 axis. Additionally, we presented preclinical data demonstrating the potential of combining gemcitabine with inhibitors targeting the macrophage-CCL5-Sp1-AREG feedback loop. Notably, inhibitors such as Gef, VP, MAR, and particularly MIT showed promising results in cell lines, PDOs, and mouse models, suggesting their potential for treating PAAD (Fig. 8).

### Supplementary Information

**Additional file 1. **Western Blotting.

**Additional file 2: Fig. S1.** Macrophages enhance the invasiveness of PCCs in the co-culture model. **Fig. S2.** Representative pictures of macrophages promoting PCCs resist gemcitabine. **Fig. S3.** 4 cytokines caused poor prognosis in pancreatic cancers. **Fig. S4.** Macrophage induced CSCs increasing in PAAD. **Fig. S5.** Macrophages promote tumor development in the in vivo model. **Fig. S6.** The CCL-5/AKT/Sp1/CD44 axis was involved in the acquisition of cancer stem-like properties in pancreatic cancer. **Fig. S7.** The concentration of IL-1β and IL-10 was measured in RAW264.7 cells after treatment with IFN-γ and LPS, as well as IL4 and IL13, respectively. **Fig. S8.** The cell viability of 6606PDA cells treated with gemcitabine at 0.25 μM and 0.0625 μM for 2 days.

## Data Availability

The datasets used and/or analyzed during the current study are available from the first author on reasonable request.

## Abbreviations

CSCs: Cancer Stem Cells
Gef: Gefitinib
Gem: Gemcitabine
GR: Gemcitabine Resistance
IC_50_: The Half-maximal Inhibitory Concentration
MAR: Maraviroc
MIT: Mithramycin
OS: Overall Survival
PDOs: Patient-Derived Organoids
PAAD: Pancreatic Adenocarcinoma
PCCs: Pancreatic Cancer Cells
PFI: Progression-Free Interval
TCGA: The Cancer Genome Atlas
TPM: Transcripts Per Million
VP: Verteporfin
