# Supplementary figures and images for: Macrophage-organoid co-culture model for identifying treatment strategies against macrophage-related gemcitabine resistance
Source: J Exp Clin Cancer Res. 2023 Aug 9;42:199. doi: 10.1186/s13046-023-02756-4 (PMC10411021; doi:10.1186/s13046-023-02756-4)

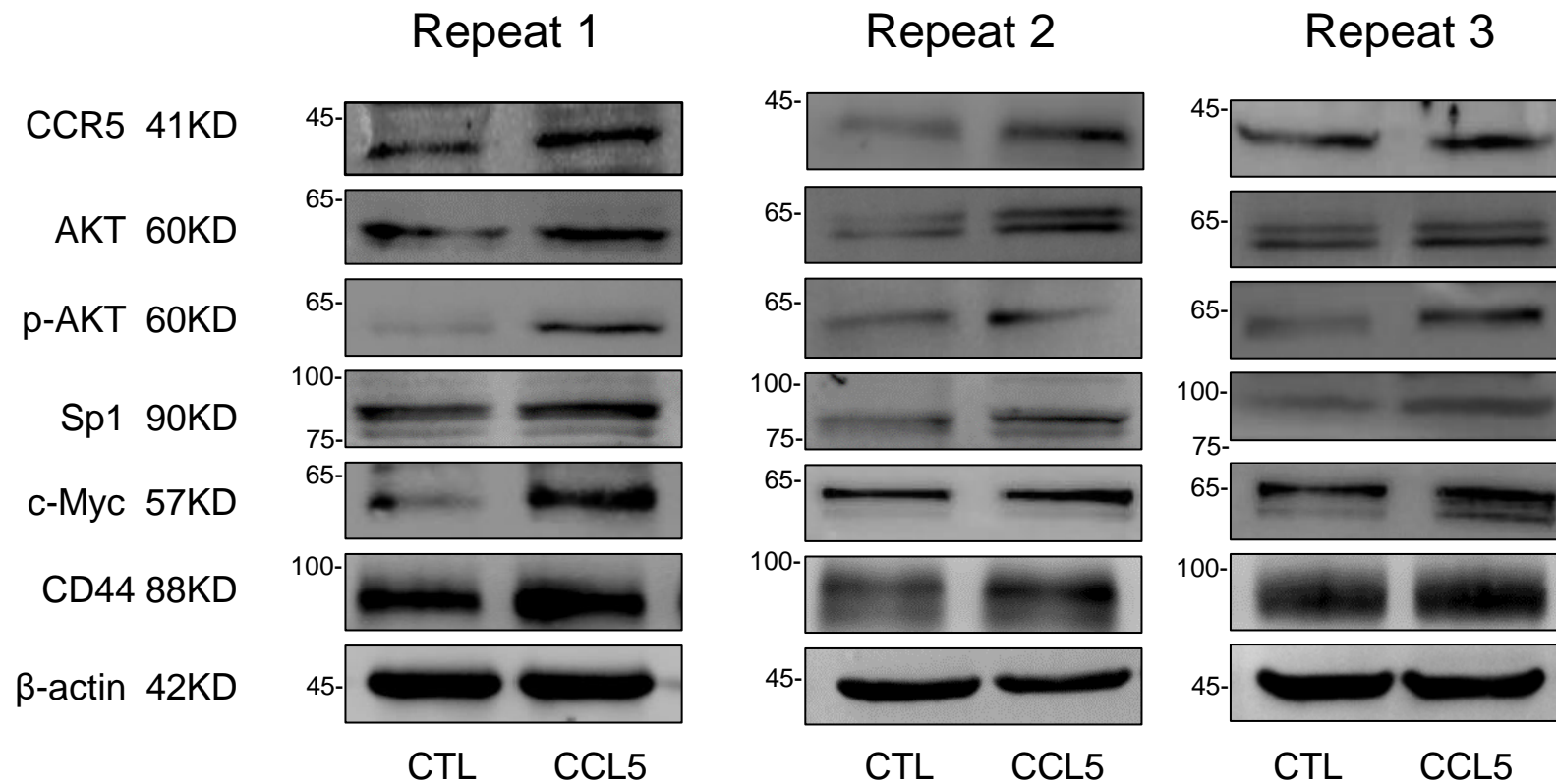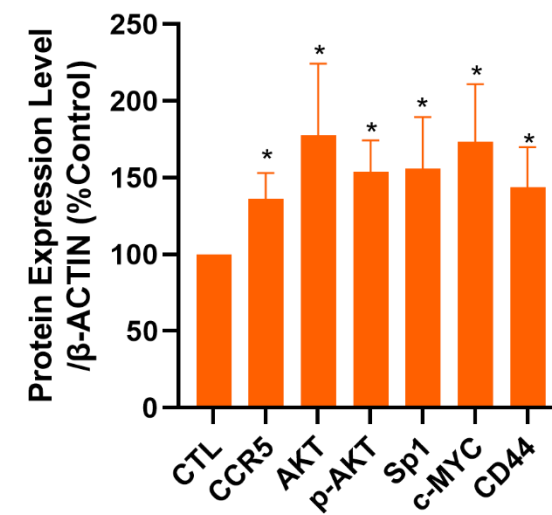

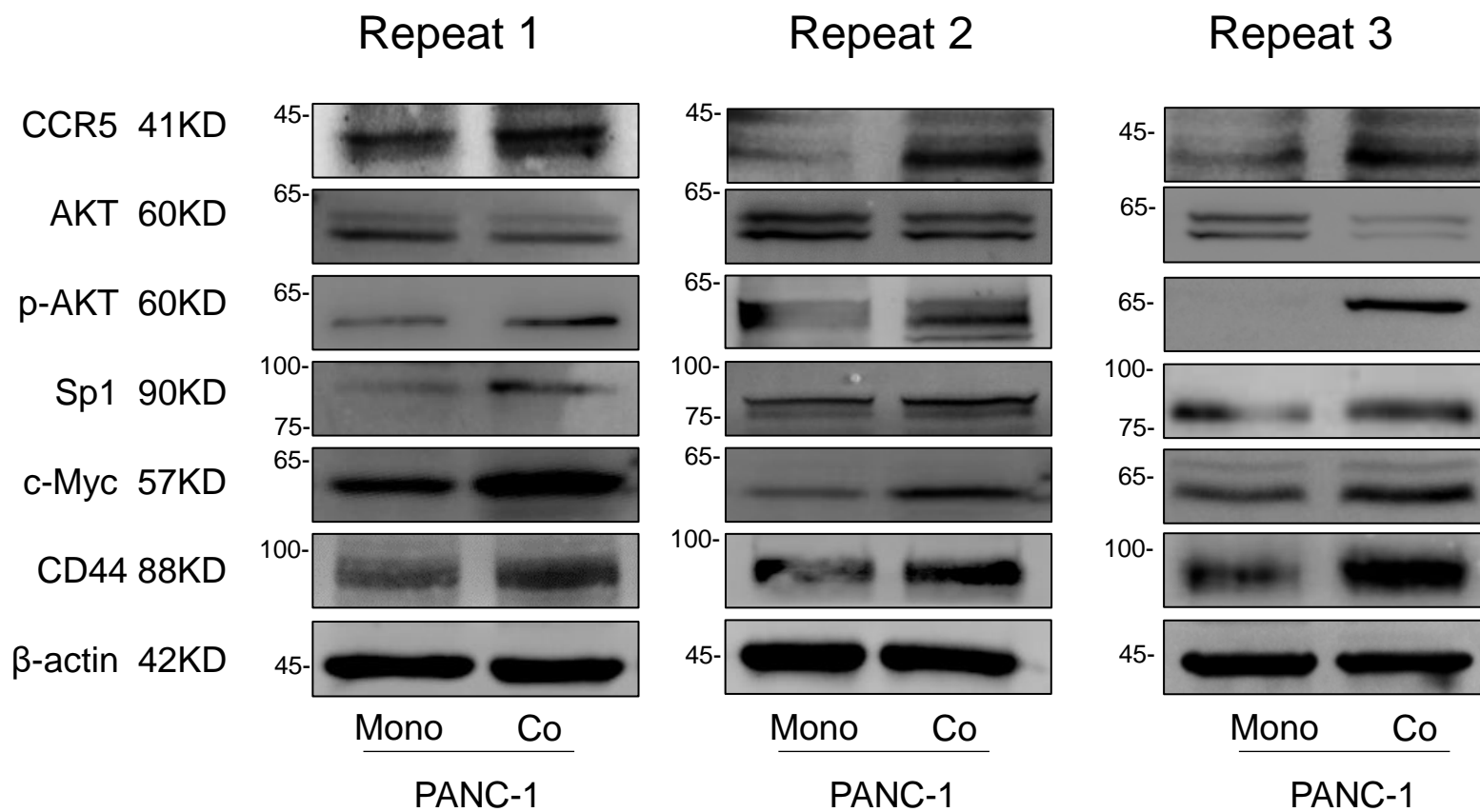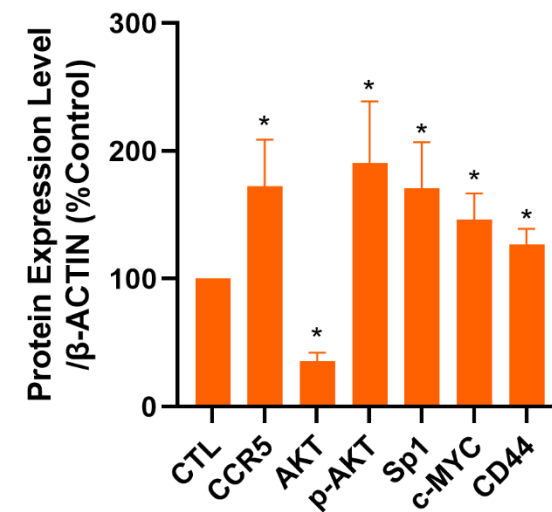

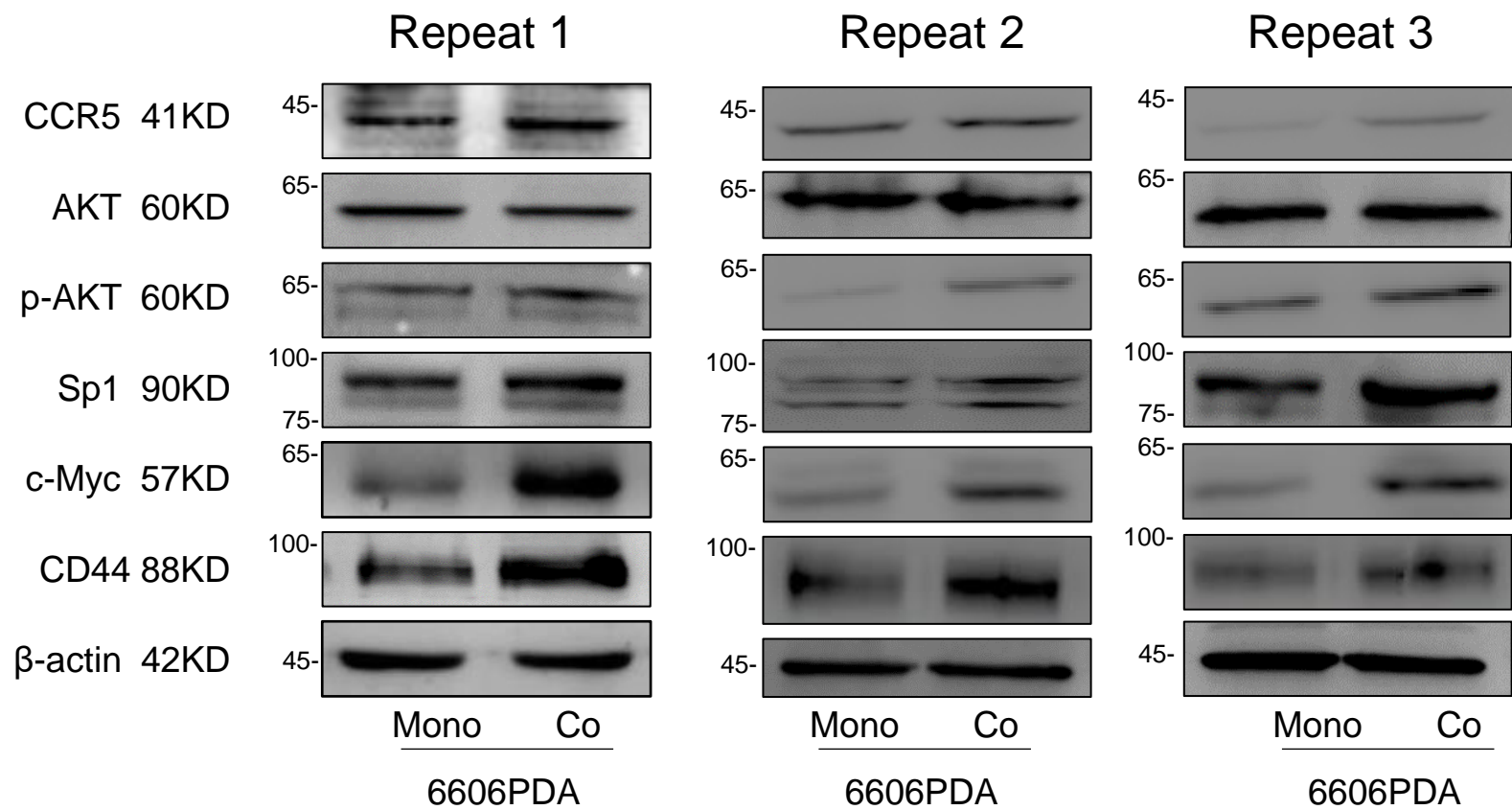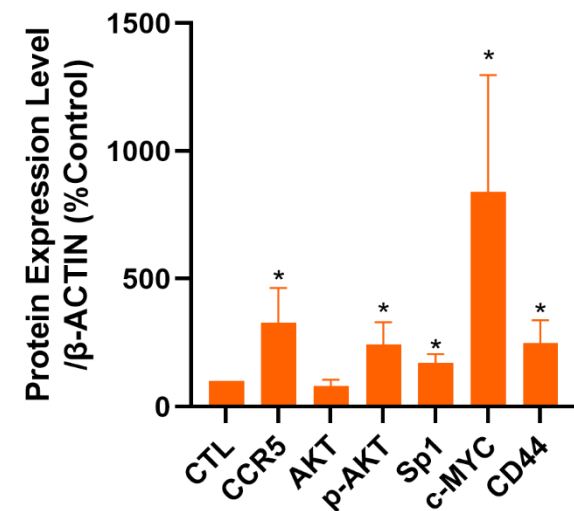

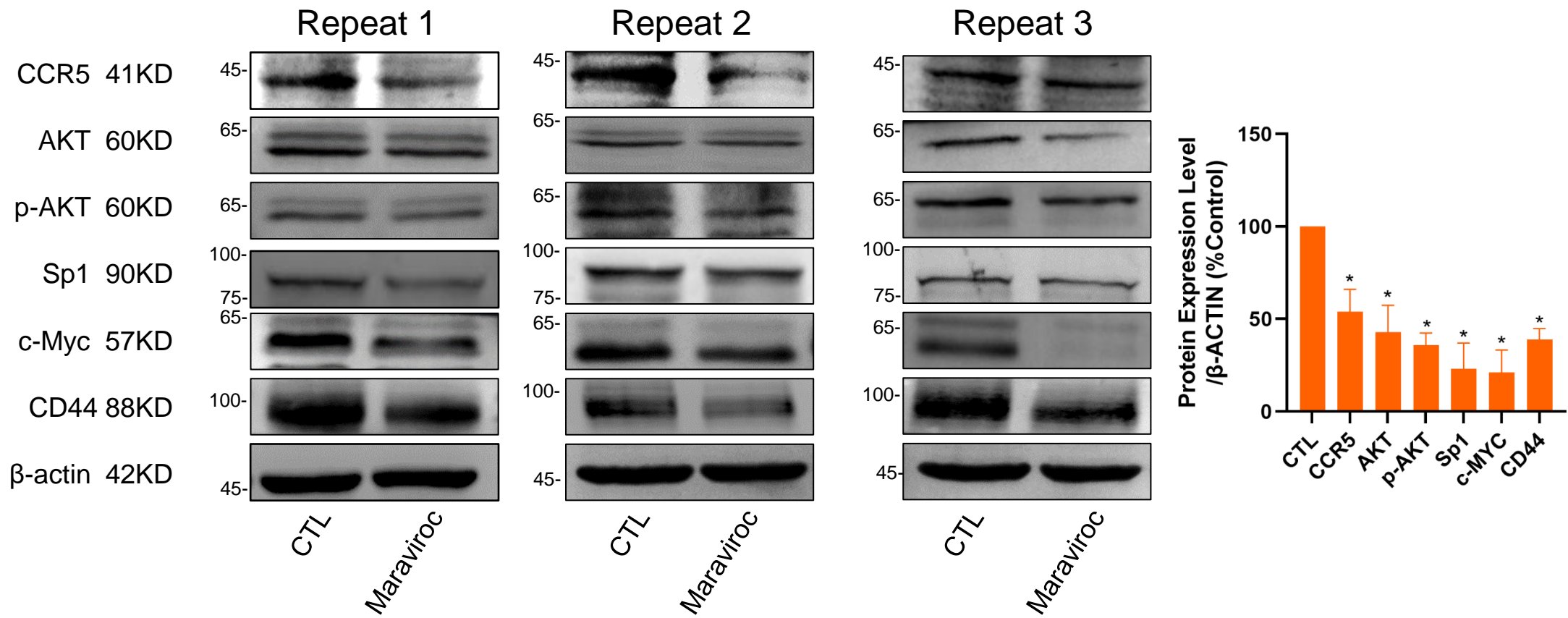

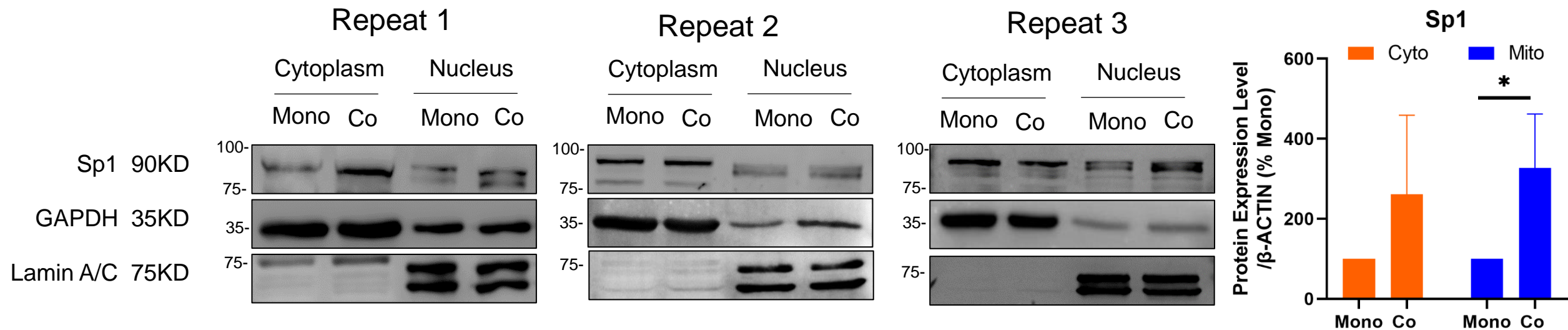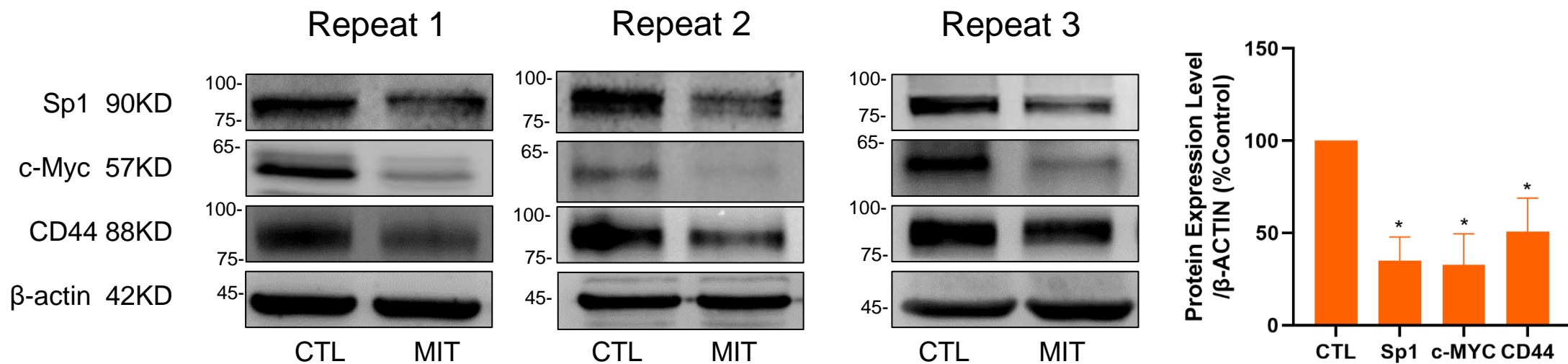

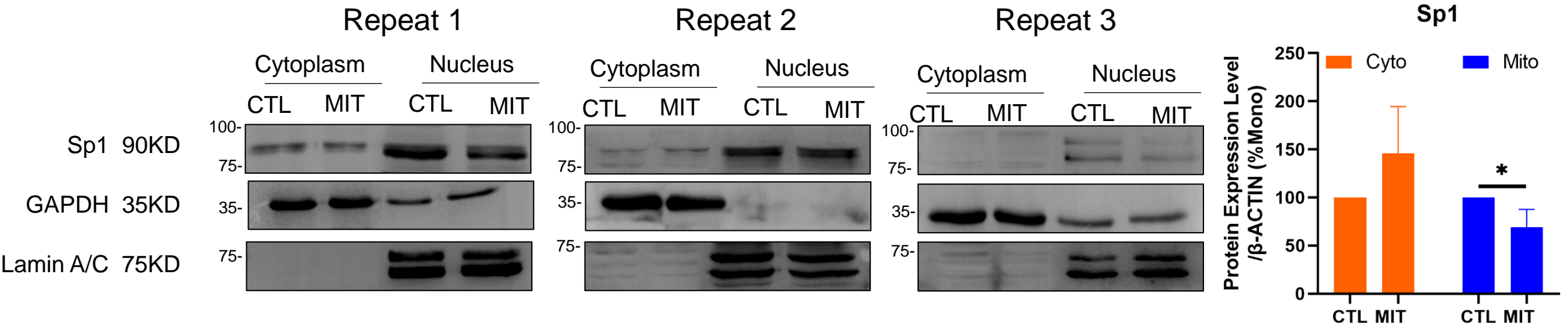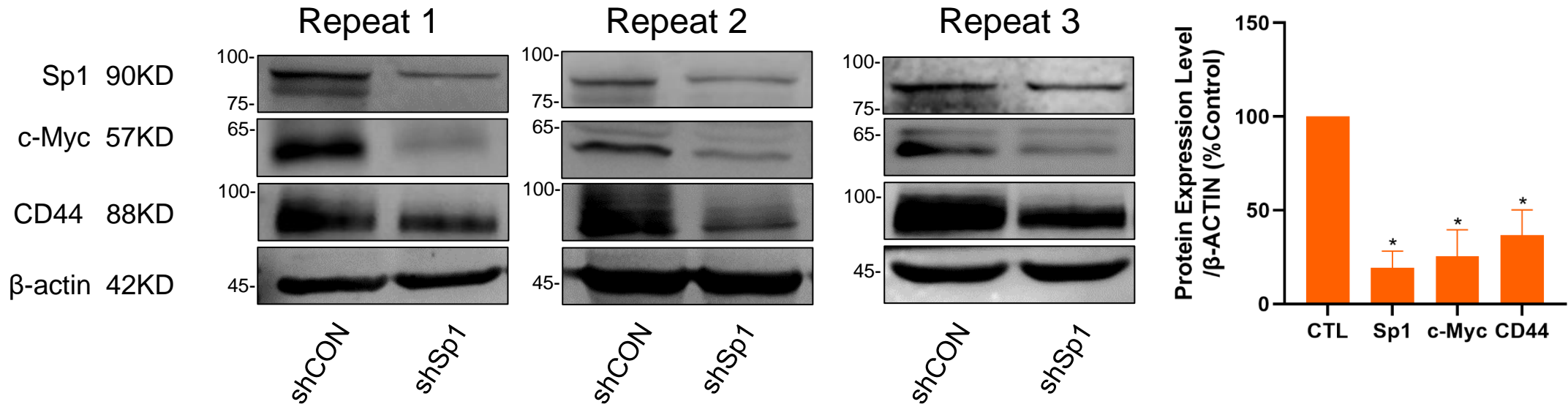

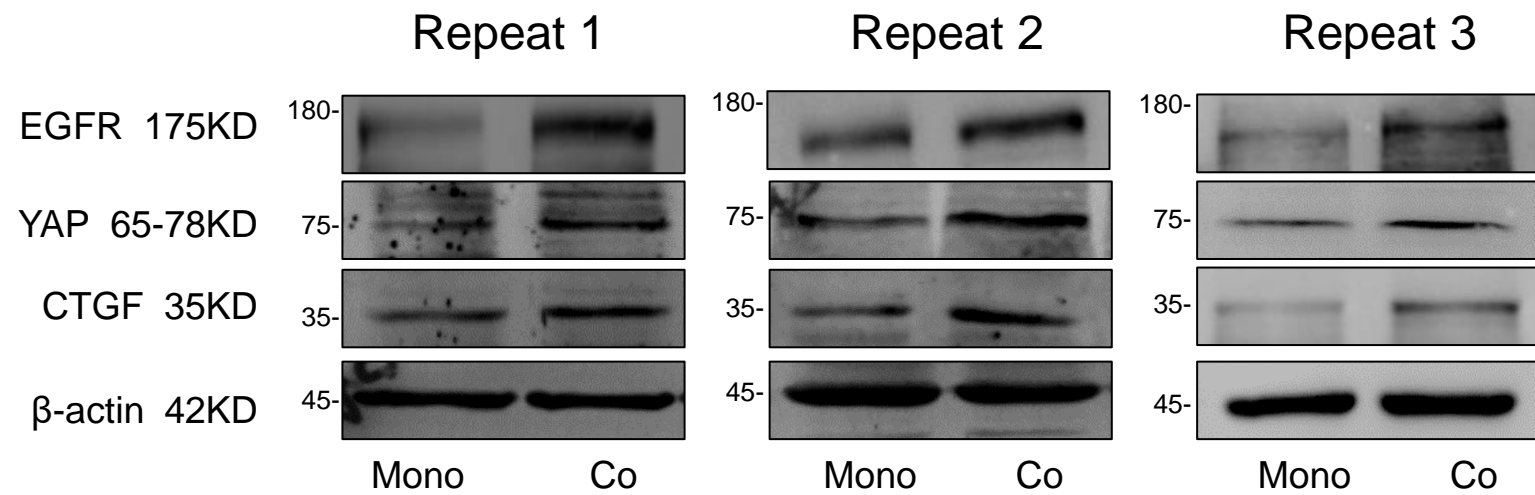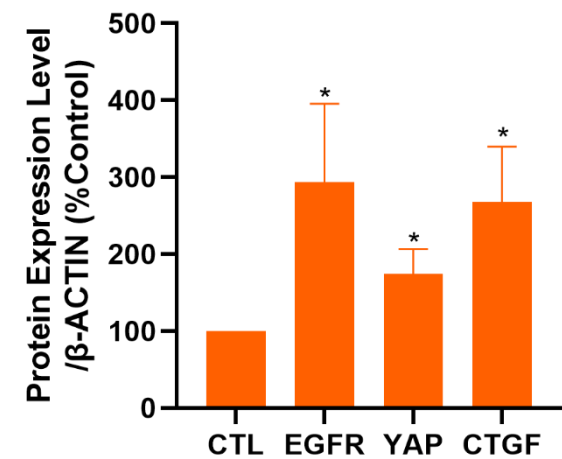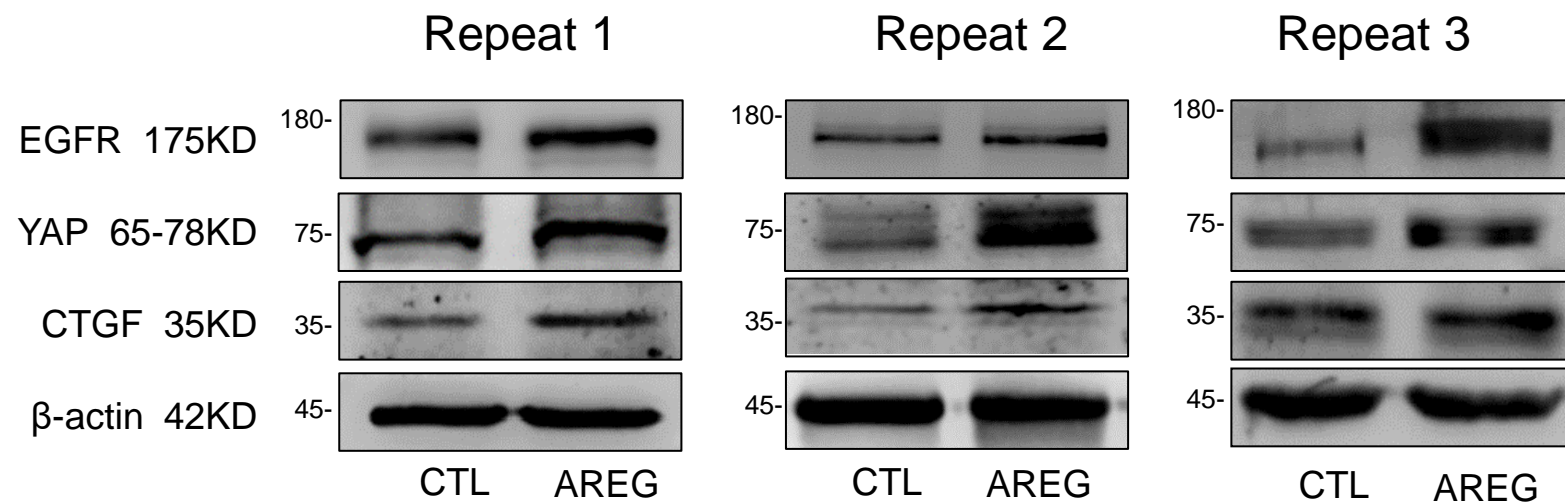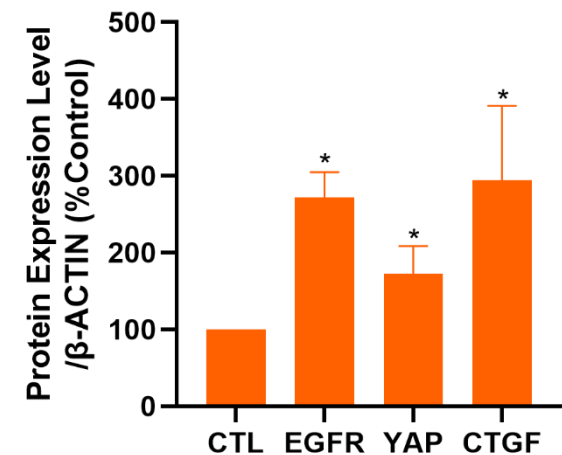

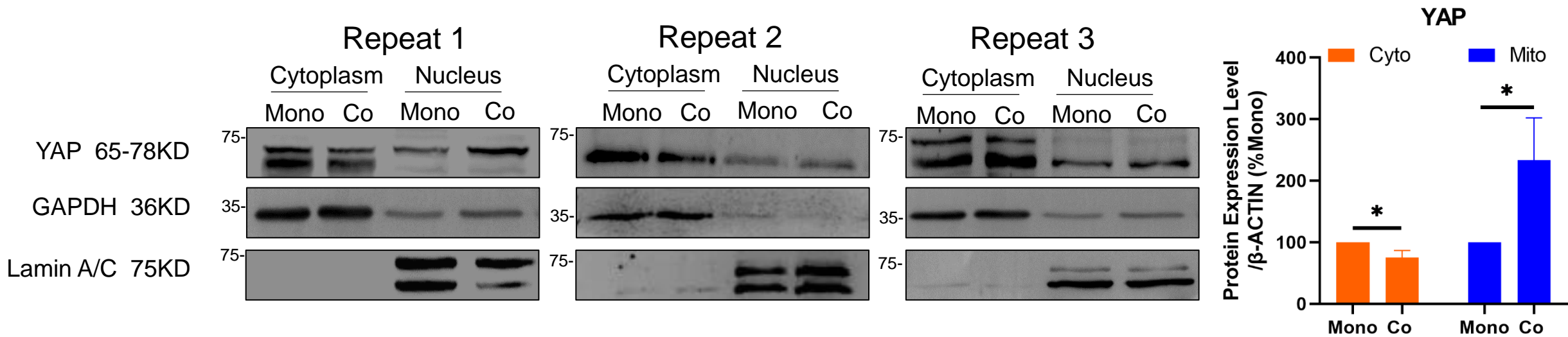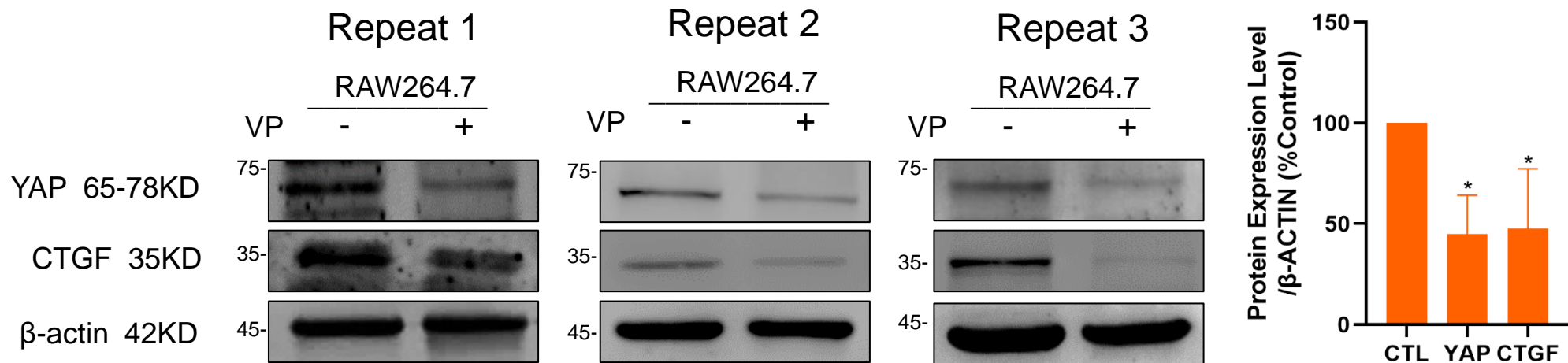

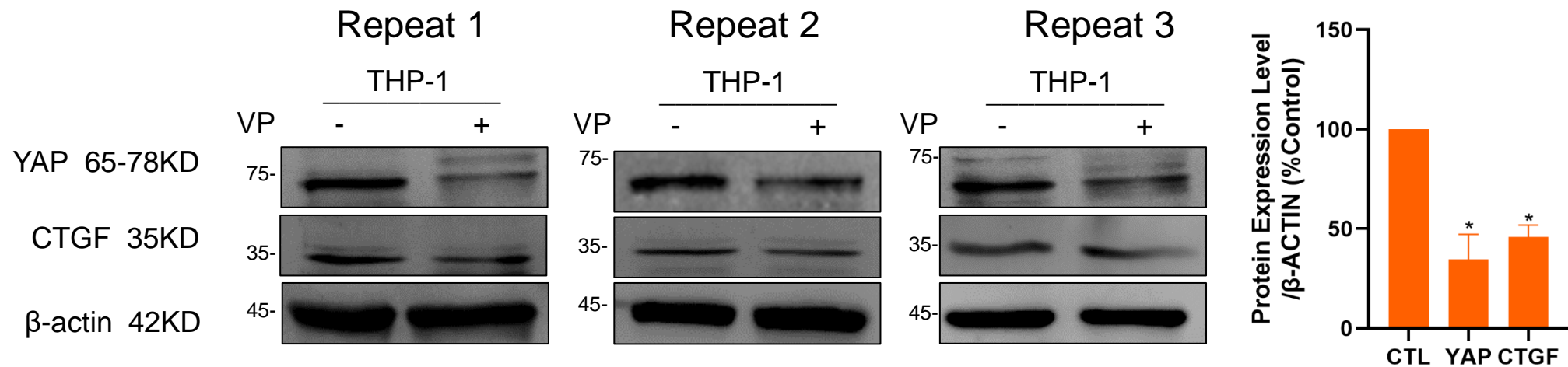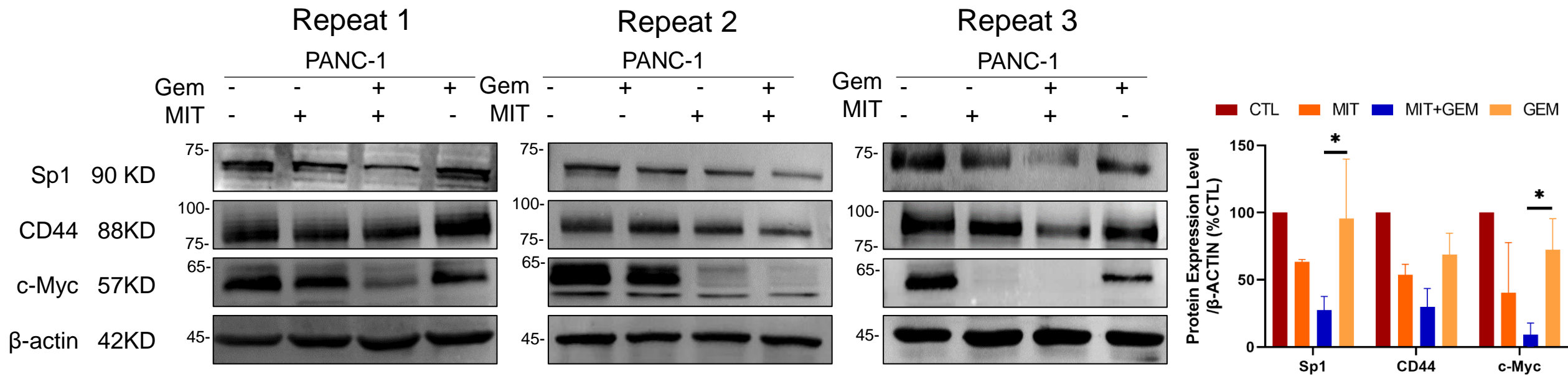

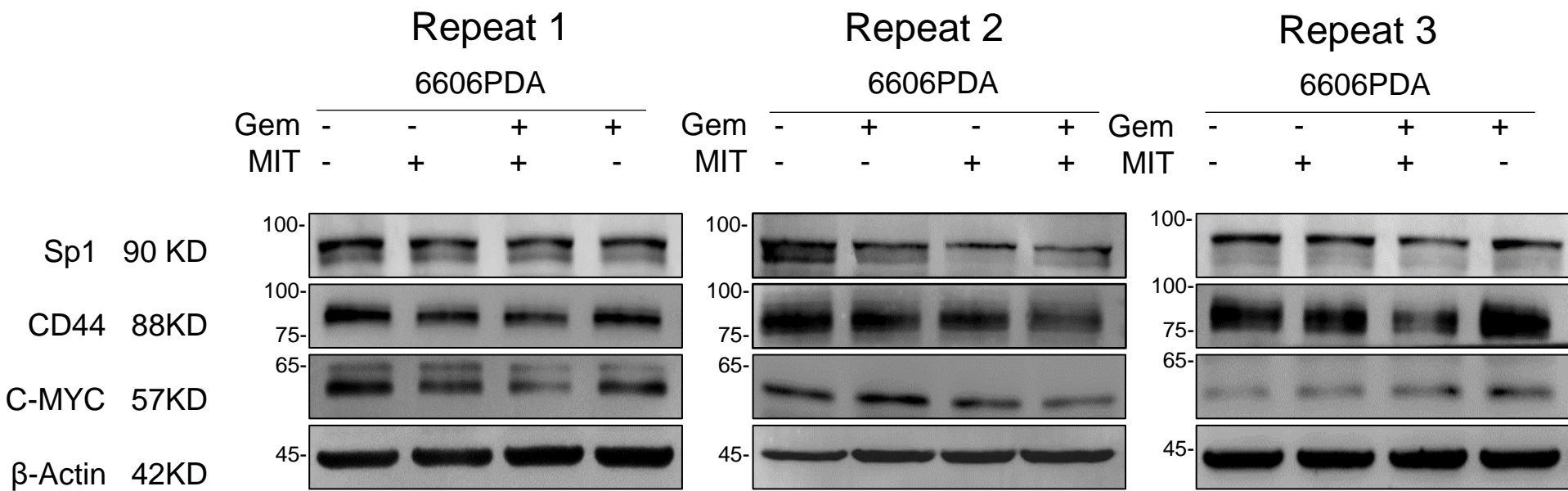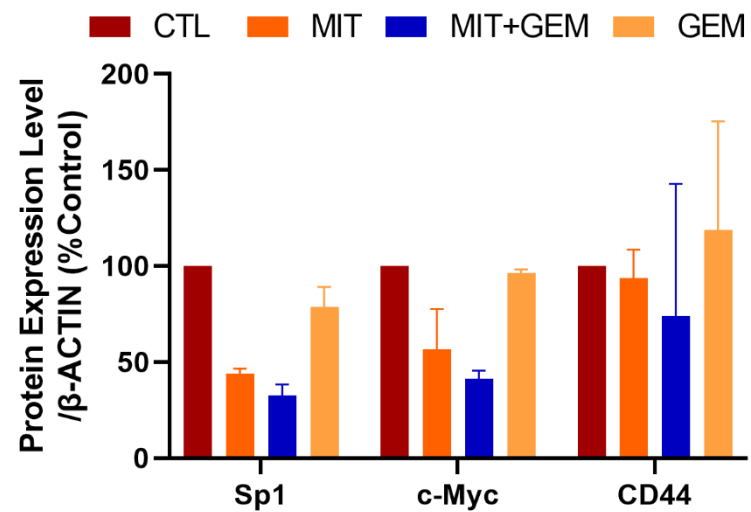

Supplement: Supplementary file 1 — Additional file 1. Western Blotting. [file 13046_2023_2756_MOESM1_ESM.pdf]
