# Supplementary material for: Macrophage-organoid co-culture model for identifying treatment strategies against macrophage-related gemcitabine resistance
Source: J Exp Clin Cancer Res. 2023 Aug 9;42:199. doi: 10.1186/s13046-023-02756-4 (PMC10411021; doi:10.1186/s13046-023-02756-4)
Supplement: Supplementary file 2 — Additional file 2: Fig. S1. Macrophages enhance the invasiveness of PCCs in the co-culture model. Fig. S2. Representative pictures of macrophages promoting PCCs resist gemcitabine. Fig. S3. 4 cytokines caused poor prognosis in pancreatic cancers. Fig. S4. Macrophage induced CSCs increasing in PAAD. Fig. S5. Macrophages promote tumor development in the in vivo model. Fig. S6. The CCL-5/AKT/Sp1/CD44 axis was involved in the acquisition of cancer stem-like properties in pancreatic cancer. Fig. S7. The concentration of IL-1β and IL-10 was measured in RAW264.7 cells after treatment with IFN-γ and LPS, as well as IL4 and IL13, respectively. Fig. S8. The cell viability of 6606PDA cells treated with gemcitabine at 0.25 μM and 0.0625 μM for 2 days. [file 13046_2023_2756_MOESM2_ESM.pdf]

Supplementary data

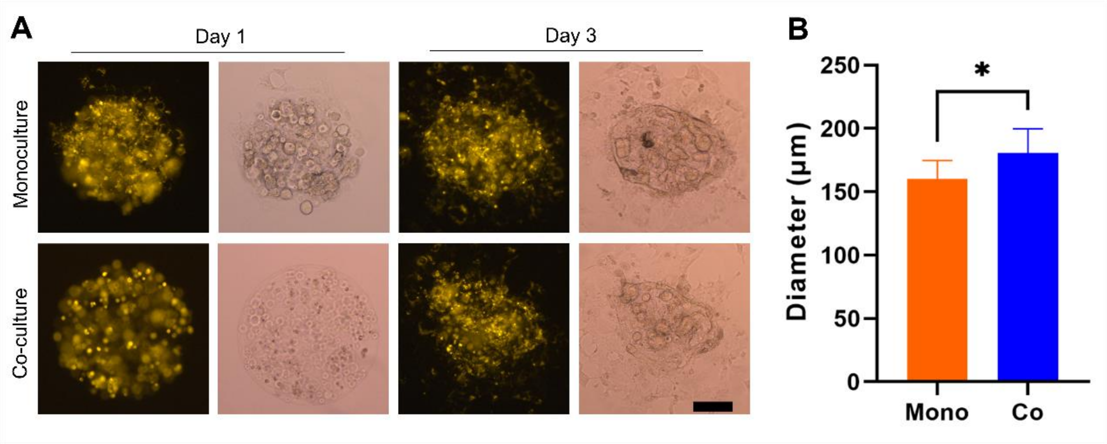

Fig. S1 Macrophages enhance the invasiveness of PCCs in the co-culture model. A Representative images depict 3D spheroids of 6606PDA cells in two different models at day 1 and day 3. **B** The diameter of 3D spheroids at day 3. (n = 10). *p<0.05.

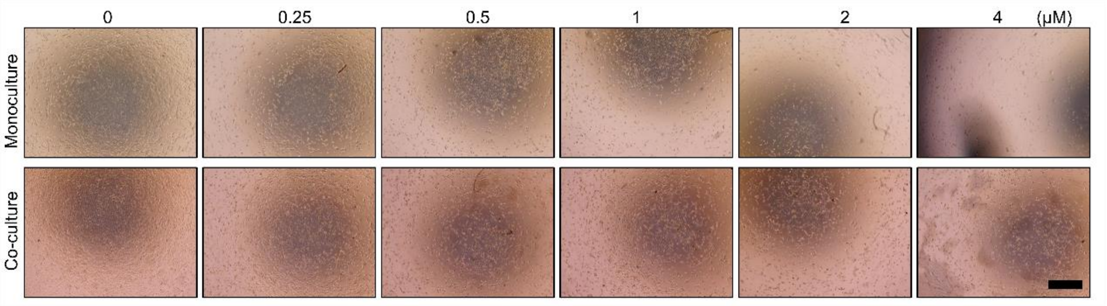

Fig. S2 Representative pictures of macrophages promoting PCCs resist gemcitabine.

Scale bar: 200 μM

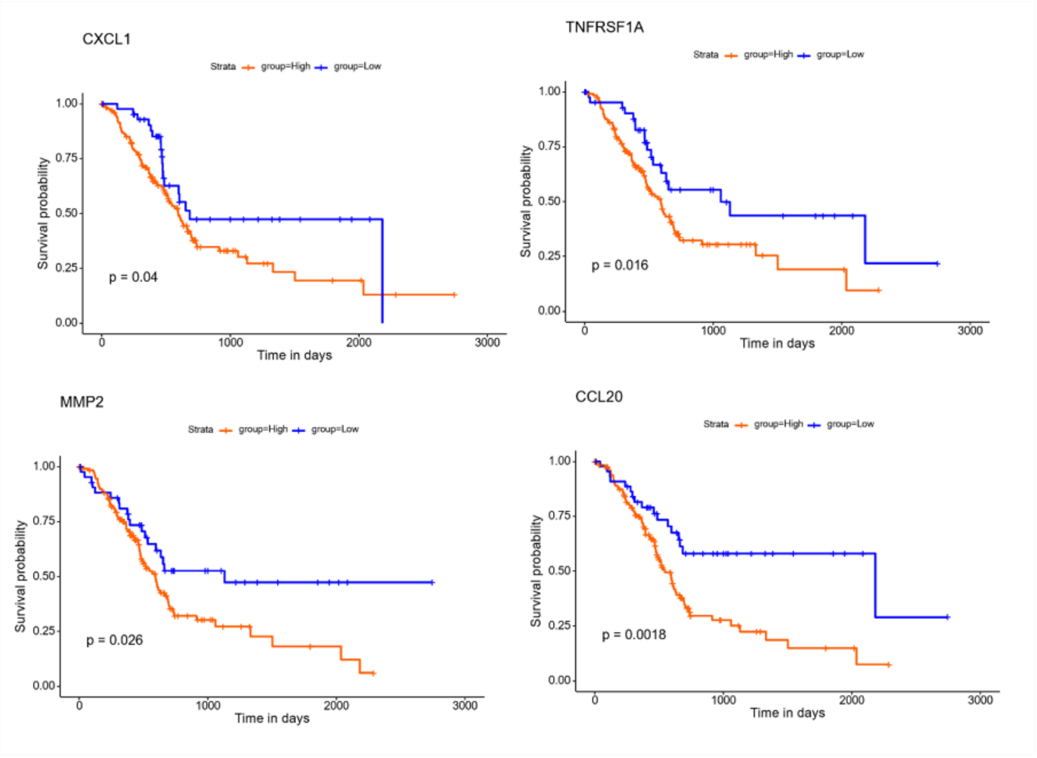

Fig. S3 4 cytokines caused poor prognosis in pancreatic cancers. Data were derived from the TCGA cohort.

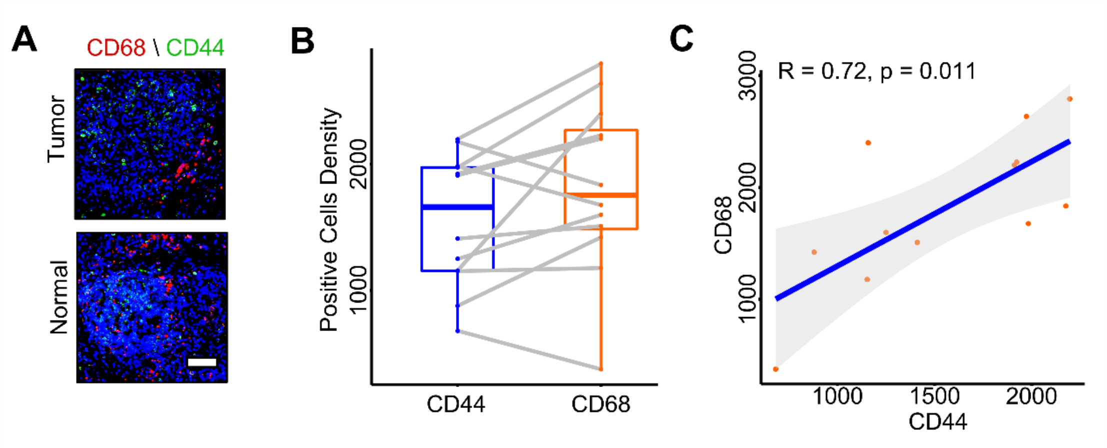

Fig. S4 Macrophage induced CSCs increasing in PAAD. A Representative picture of PAAD tumor and para-tumor stained by CD68 and CD44. **B** Positive cell density analysis of each immunohistochemistry image of CD68 and CD44. (n = 12). **C** Spearman's correlation analysis of CD44 and CD68 in pancreatic cancer tissues depends on positive

cell density. (n = 12).

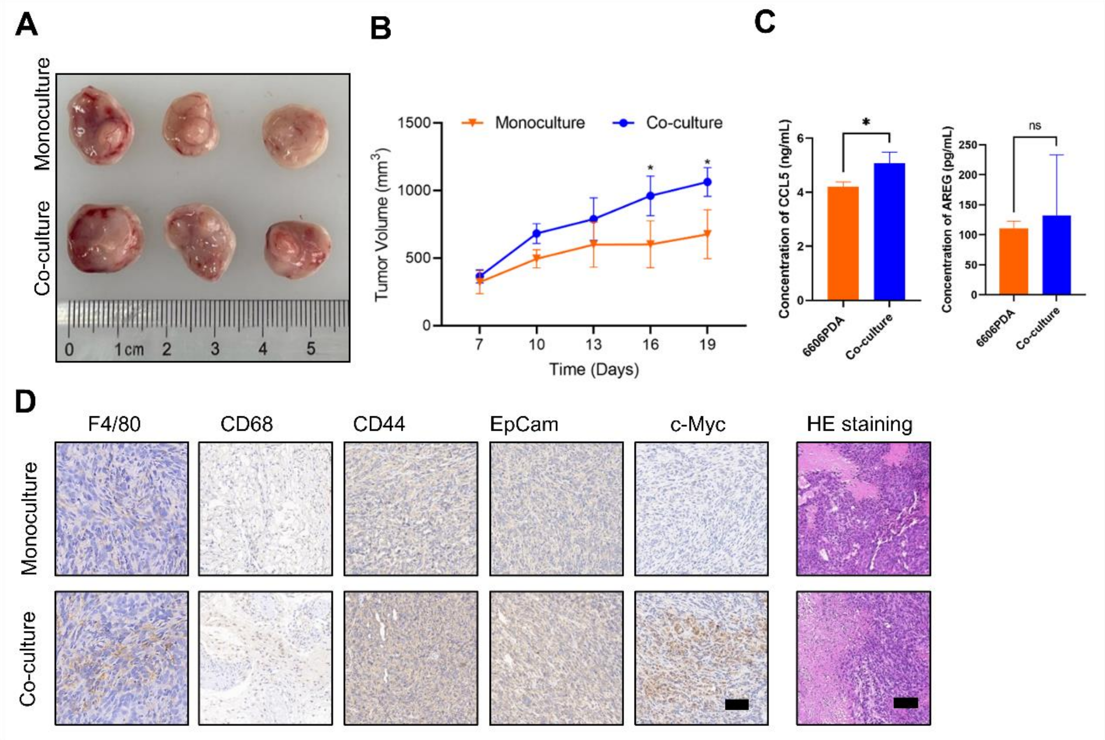

Fig. S5 Macrophages promote tumor development in the *in vivo* model. A The tumor images were in two different groups. 6606PDA cells co-culture with RAW264.7 cells for two weeks and then transplanted into nude mice subcutaneously. **B** The changes in tumor volume in two groups (n=3). **C** The quantitative analysis of mouse serum's AREG (right) and CCL5 (left) in two models at day 19 after transplant. (n = 3). **D** The representative immunohistochemistry images of F4/80, CD68, CD44, c-Myc, or EpCam. Scale bar: 50 μm. The hematoxylin-eosin (HE) staining images. Scale bar: 50 μm. ns = no signicant, *p<0.05.

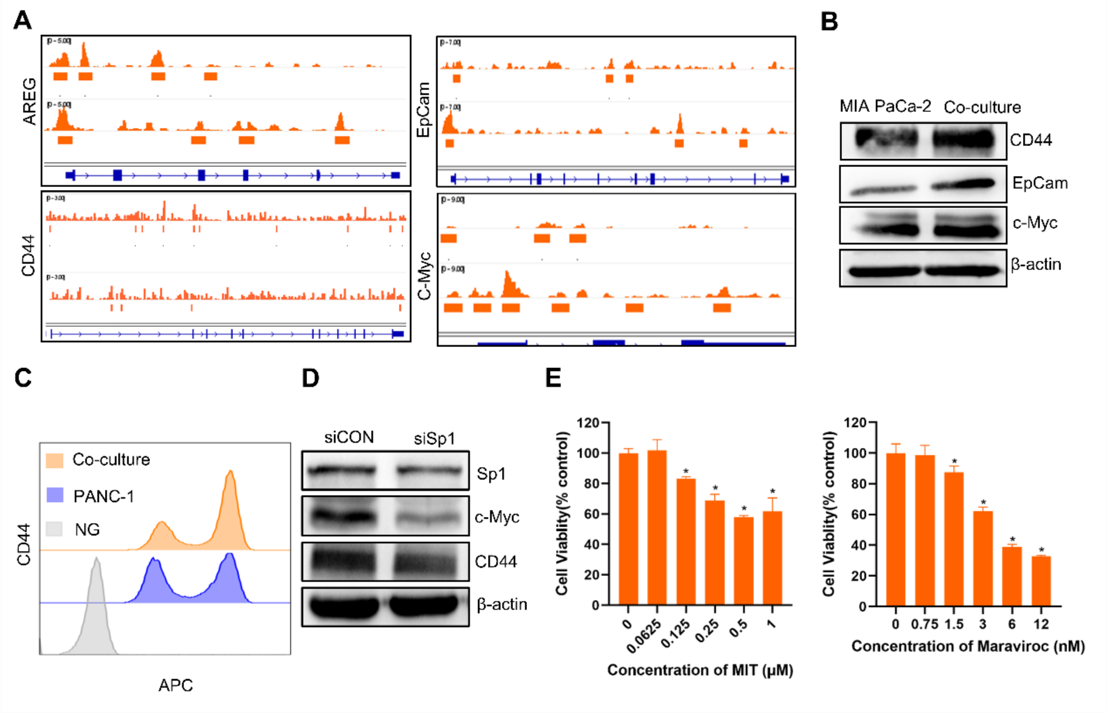

Fig. S6 The CCL-5/AKT/Sp1/CD44 axis was involved in the acquisition of cancer stem-like properties in pancreatic cancer. A Sp1 regulated the expression of AREG, CD44, EpCam, and c-MYC. Data were obtained from the Gene Expression Omnibus (GEO) dataset (GSM2424246, GSM2424247). **B** Protein expression of MIA PaCa-2 cell lines. **C** Flow cytometry analysis of CD44 expression. **D** siSp1 inhibited the protein expression of CD44 and c-Myc. **E** Cell viability of PANC-1 cells treated with MIT (left) or Maraviroc (right) for 2 days (n = 3). *p <0.05.

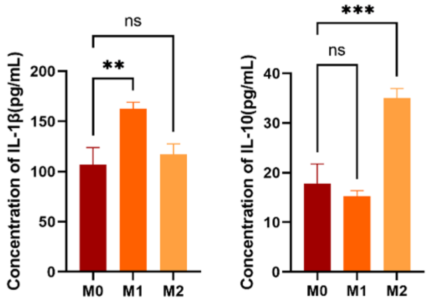

Fig. S7 The concentration of IL-1β and IL-10 was measured in RAW264.7 cells after

treatment with IFN-γ and LPS, as well as IL4 and IL13, respectively. (n = 3). ns = no significant, **p <0.01, ***p <0.001.

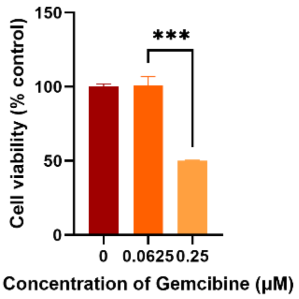

Fig. S8 The cell viability of 6606PDA cells treated with gemcitabine at 0.25 μM and 0.0625 μM for 2 days. ***p <0.001.
